# Nano-laponite encapsulated coaxial fiber scaffold promotes endochondral osteogenesis

**DOI:** 10.1093/rb/rbae080

**Published:** 2024-07-04

**Authors:** Li Yuan, Jiawei Wei, Shiqi Xiao, Shue Jin, Xue Xia, Huan Liu, Jiangshan Liu, Jiaxin Hu, Yi Zuo, Yubao Li, Fang Yang, Jidong Li

**Affiliations:** Research Center for Nano-Biomaterials, Analytical and Testing Center, Sichuan University, Chengdu 610064, China; Research Center for Nano-Biomaterials, Analytical and Testing Center, Sichuan University, Chengdu 610064, China; Research Center for Nano-Biomaterials, Analytical and Testing Center, Sichuan University, Chengdu 610064, China; Research Center for Nano-Biomaterials, Analytical and Testing Center, Sichuan University, Chengdu 610064, China; Research Center for Nano-Biomaterials, Analytical and Testing Center, Sichuan University, Chengdu 610064, China; Research Center for Nano-Biomaterials, Analytical and Testing Center, Sichuan University, Chengdu 610064, China; Research Center for Nano-Biomaterials, Analytical and Testing Center, Sichuan University, Chengdu 610064, China; Research Center for Nano-Biomaterials, Analytical and Testing Center, Sichuan University, Chengdu 610064, China; Research Center for Nano-Biomaterials, Analytical and Testing Center, Sichuan University, Chengdu 610064, China; Research Center for Nano-Biomaterials, Analytical and Testing Center, Sichuan University, Chengdu 610064, China; Department of Dentistry—Regenerative Biomaterials, Research Institute for Medical Innovation, Nijmegen, 6525EX, The Netherlands; Research Center for Nano-Biomaterials, Analytical and Testing Center, Sichuan University, Chengdu 610064, China

**Keywords:** coaxial electrospinning, laponite RDS sol, endochondral ossification, bone regeneration

## Abstract

Osteoinductive supplements without side effects stand out from the growth factors and drugs widely used in bone tissue engineering. Lithium magnesium sodium silicate hydrate (laponite) nanoflake is a promising bioactive component for bone regeneration, attributed to its inherent biosafety and effective osteoinductivity. Up to now, the *in vivo* osteogenic potential and mechanisms of laponite-encapsulated fibrous membranes remain largely unexplored. This study presents a unique method for homogeneously integrating high concentrations of laponite RDS into a polycaprolactone (PCL) matrix by dispersing laponite RDS sol into the polymer solution. Subsequently, a core-shell fibrous membrane (10RP-PG), embedding laponite-loaded PCL in its core, was crafted using coaxial electrospinning. The PCL core’s slow degradation and the shell’s gradient degradation enabled the sustained release of bioactive ions (Si and Mg) from laponite. *In vivo* studies on a critical-sized calvarial bone defect model demonstrated that the 10RP-PG membrane markedly enhanced bone formation and remodeling by accelerating the process of endochondral ossification. Further transcriptome analysis suggested that osteogenesis in the 10RP-PG membrane is driven by Mg and Si from endocytosed laponite, activating pathways related to ossification and endochondral ossification, including Hippo, Wnt and Notch. The fabricated nanocomposite fibrous membranes hold great promise in the fields of critical-sized bone defect repair.

## Graphical Abstract

**Figure:**
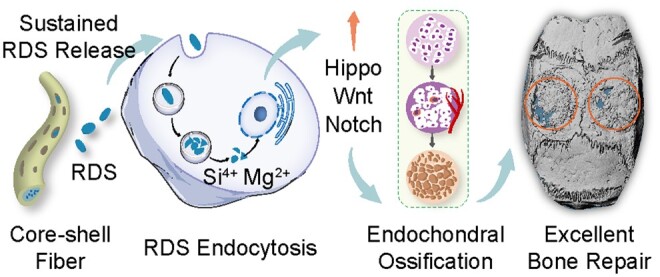

## Introduction

Natural bone possesses a remarkable self-healing capacity after injuries and fractures. However, repairing large, critical-sized bone defects remains a significant clinical challenge [1]. Bone tissue engineering scaffolds are viewed as ideal substitutes for bone grafts. Among these, electrospun fibrous scaffolds have garnered widespread attention due to their resemblance to the extracellular matrix structure [2]. The ideal bone scaffold design should not only exhibit structural integrity but also include safe, bioactive agents that promote osteoinduction, stimulating bone growth and enhancing osseointegration without adverse effects.

While integrating growth factors such as BMP-2 into bone repair scaffolds has demonstrated effectiveness in enhancing bone regeneration and repair, challenges like high costs, easy inactivation and potential side effects hinder their wide application [3–5]. Consequently, inorganic ions and their stable, cost-effective compounds with biological functions have drawn significant research interest [6–9]. For instance, silicate, found naturally in mammalian bone tissue, regulates collagen synthesis, mineralization of bone matrix, and various osteogenic processes [10]. Laponite (${\text{Na}}_{0.7}^{+}$[(Si_8_Mg_5.5_Li_0.3_)O_20_(OH)_4_]_0.7_^−^), a type of nanosilicate, has been extensively studied in regenerative medicine for its osteoinductive properties derived from dissolution products like Mg^2+^, Li^+^ and Si(OH)_4_ [11–14]. Magnesium ions (Mg^2+^), for example, have been reported to promote osteoblast adhesion and ossification [15, 16]. Lithium ions (Li^+^) activated the Wnt/β-catenin signaling pathway, thereby promoting cell proliferation, matrix mineralization and osteogenic differentiation [17]. Orthosilicic acid (Si(OH)_4_) stimulated osteogenic differentiation and collagen type I synthesis [18].

Previous studies have incorporated laponite into polymers to create electrospun fibrous scaffolds. For example, Zhang *et al.* [19] added laponite XLG powder to a PCL solution, later fabricating a nanofiber membrane by electrospinning after thorough stirring and ultrasonic treatment. This membrane demonstrated its ability to promote cell adhesion, osteogenic differentiation *in vitro* and even ectopic bone formation *in vivo*. Similarly, Shi *et al.* [20] introduced laponite particles into a PLGA solution under stirring, subsequently achieving a laponite-doped PLGA nanofiber membrane by electrospinning. However, directly adding laponite into other solution systems in these studies may disrupt its dispersion, leading to inconsistent distribution and potential sedimentation, especially at high concentrations. Such inhomogeneous dispersion can clog the spinning needle during electrospinning, compromising the mechanical integrity of the fibrous membrane. Therefore, developing new strategies for achieving high and uniform laponite distribution in electrospun scaffolds is crucial to further explore laponite’s osteogenic effects and potential mechanisms *in vivo*.

To address the challenge of achieving stable and uniform dispersion of laponite particles in a polymer matrix, various laponite products and incorporation methods were explored. Two primary laponite products were selected: gel-grade laponite (RD) and sol-grade laponite (RDS). At a 3% concentration, gel-grade laponite quickly forms a high-viscosity pre-gel in water. Sol-grade laponite, with a small amount of tetrasodium pyrophosphate, inhibits thixotropic gel formation. Remarkably, even at 30% concentration, low-viscosity sols are achievable. Subsequently, both direct incorporation and aqueous dispersion incorporation methods were utilized to evaluate the dispersibility of laponite in solutions. We opted for polycaprolactone (PCL), a versatile biopolymer approved by the FDA for biomedical applications, to house laponite. PCL forms a morphologically stable fibrous scaffold, resisting shrinkage and degrading slowly. However, PCL’s hydrophobicity does not favor cell attachment. To address this, we devised a coaxial electrospinning strategy where PCL forms the core, while a blend of PLGA (providing adjustable degradation) and gelatin (noted for superior biocompatibility) constitutes the shell. Coaxial electrospinning has been proven to control the release of drugs in the core [21]. This core-shell configuration leverages the slow degradation of PCL to ensure a prolonged and stable release of laponite. Concurrently, the rapid degradation of gelatin within the shell creates channels, facilitating the release of laponite components. This design preserves the scaffold’s structure integrity while enabling controlled laponite release.

**Scheme 1.**
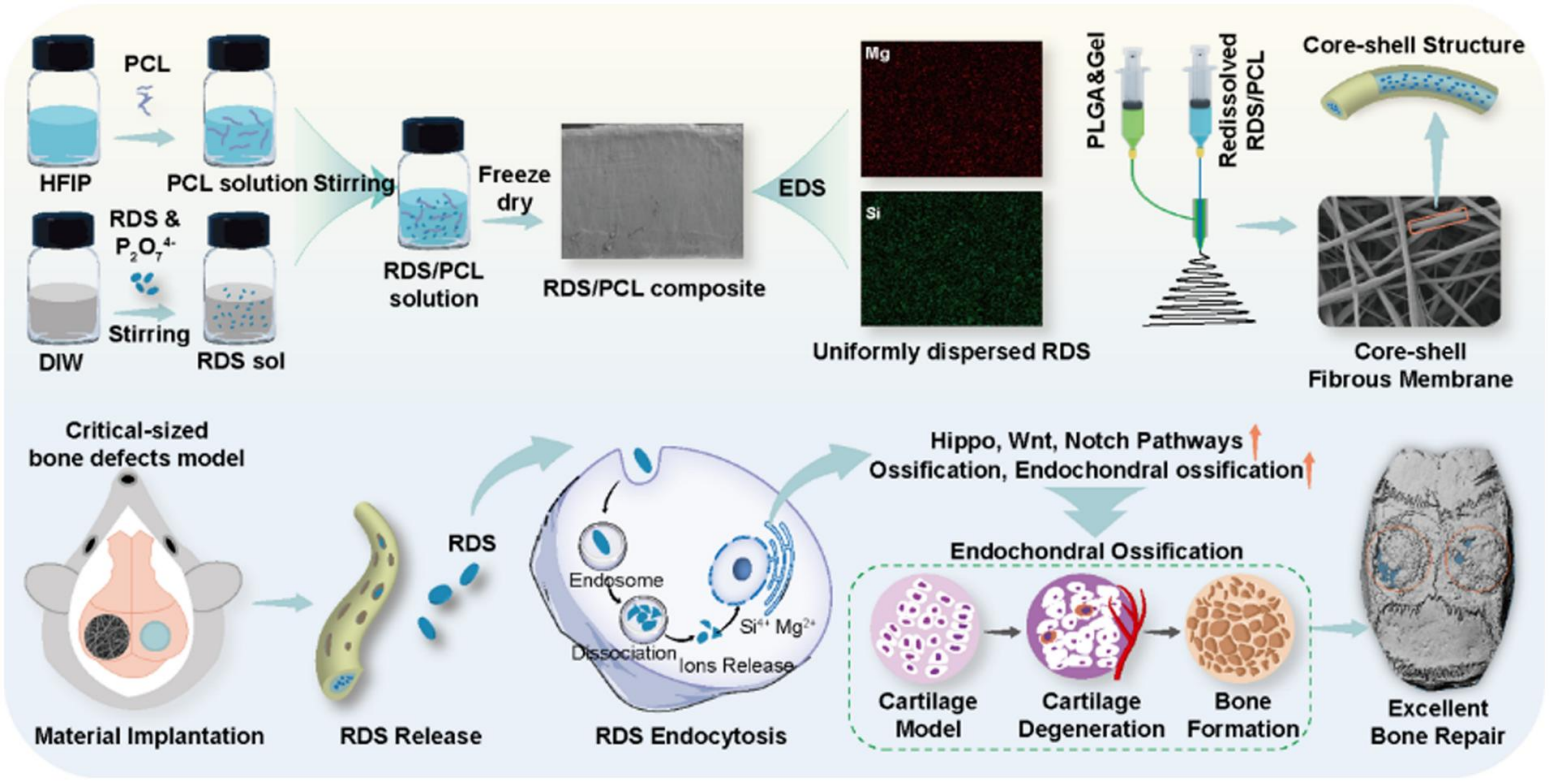
Illustration of the preparation process of nano-laponite encapsulated core-shell fiber scaffold and its application in bone regeneration.

We studied the dispersibility and spinnability of laponite in core-spinning solution and assessed core-shell fibrous membranes for shrinkage resistance, mechanical properties, degradation and ion release characteristics (Scheme 1). In addition, we systematically evaluated the *in vitro* and *in vivo* biocompatibility and osteogenic properties of these membranes. A critical-sized skull defect model assisted us in determining the effect of composite fibrous membrane on guided bone regeneration, and we delved into the possible osteogenesis mechanism via transcriptome analysis.

## Experimental

### Materials and equipment

Poly(lactic-co-glycolic acid) (PLGA, LA/GA ratio = 85:15, Mw = 300 kDa), PCL (Mw = 80 kDa), RD, RDS, gelatin (B-type gelatin derived from bovine bones, with molecular weights ranging from tens of thousands to several hundred thousand) and 1,1,1,3,3,3-hexafluoro-2-isopropanol (HFIP) were purchased for the fabrication of fibrous membranes. The materials and equipment used in this study are shown in Supplementary Tables S1 and S2, respectively.

### Optimization of preparation method

Direct incorporation method: Dissolve PCL into HFIP to obtain a 12% PCL (w/v) solution. Gel-grade laponite (RD) and sol-grade laponite (RDS) were added to PCL solutions, respectively and stirred magnetically for 24 h to prepare 10% RD/PCL and 10% RDS/PCL solutions.

Aqueous dispersion incorporation method: RD and RDS powders were added to deionized water (DIW) under magnetic stirring to prepare 2% RD, 5% RD, 10% RDS and 20% RDS solutions (w/v), magnetic stirring for 24 h, and then stand for 12 h. The obtained aqueous dispersions were added to PCL solutions, respectively and stirred for 24 h to prepare 10% RD/PCL and 10% RDS/PCL solutions.

Ultimately, a 10% RDS sol was selected for preparing various concentrations of RDS/PCL solutions. To assess the RDS sol incorporation method across different polymer matrices, 10% RDS/PLGA, RDS/CS (chitosan) and RDS/PU (polyurethanes) solutions were prepared and characterized.

RDS/PCL solutions were lyophilized for 72 h and then redissolved in HFIP (12% w/v) for electrospinning core solutions. For the shell layer, gelatin and PLGA mixtures, in ratios ranging from 5:100–20:100, were dissolved in HFIP (12% w/v) with overnight magnetic agitation. To explore spinnability, RDS/PCL (RP) and PLGA/Gel (PG) uniaxial fibrous membranes were fabricated. RP-PG core-shell nanofibers were fabricated using a coaxial needle (inner 25G, outer 18G). To optimize the preparation process, membranes were electrospun at different shell/core injection ratios (1:1, 2:1 and 3:1). The electrospinning parameters for uniaxial fibrous membranes were set as follows: a flow rate of 0.1 mm/min, a high voltage of 8 kV, and a tip-to-collector distance of 15 cm. For coaxial fibrous membranes, the shell and core solution flow rates were set at 0.15 and 0.15 mm/min, 0.2 and 0.1 mm/min, 0.225 and 0.075 mm/min, respectively, along with a high voltage of 12 kV and a tip-to-collector distance of 15 cm.

Post-fabrication, membranes were vacuum-dried for complete solvent evaporation. The composition and abbreviations of the fibrous membranes used in this study are detailed in Tables 1–3. Subsequently, these membranes were cut into 10 mm discs and immersed in DI water, phosphate buffer saline (PBS) solution, 75% ethanol solution and α-MEM culture medium. After 24 h of soaking at 37°C with agitation, membrane morphology and longest diameter were analyzed.

**Table 1.** Component ratio and abbreviation of RDS and PCL blend uniaxial membrane

Abbreviation	Composition (RDS: PCL)
5RP	5: 95
10RP	10:90
20RP	20:80

**Table 2.** Component ratio and abbreviation of gel and PLGA blend uniaxial membrane

Abbreviation	Composition	Gel ratio to PLGA (w/w)
5PG	Gel, PLGA	5%
10PG	Gel, PLGA	10%
15PG	Gel, PLGA	15%
20PG	Gel, PLGA	20%

**Table 3.** Composition and abbreviation of coaxial membrane

Abbreviation	Core layer	Shell layer (w/w)
P-PG	PCL	10PG
5RP-PG	5RP	10PG
10RP-PG	10RP	10PG
20RP-PG	20RP	10PG

### Characterization of RDS and nanofibrous membranes

TEM examined the dispersion of RDS, the core-shell structure of the coaxial fibers and the presence of RDS in of 10RP and 10RP-PG nanofibers. The morphology of the RD/PCL and RDS/PCL solution smear was observed with an inverted microscope. SEM analyzed the morphology of various samples including RDS, RDS sol-lyophilized powders, RD/PCL and RDS/PCL films, and both uniaxial and coaxial fibrous membranes. EDS determined the RDS distribution in RD/PCL and RDS/PCL films and in 10RP-PG fibers. Fiber diameter distribution was analyzed using Image J software. The crystallization changes of RDS, before and after dissolution and redrying, were analyzed using XRD. XPS characterized RDS surface composition. FTIR was utilized to confirm the incorporation of RDS and Gelatin. For visualizing the core-shell structure, the shell and core electrospun solutions were mixed with Coumarin-6 and Rhodamine B, respectively, and examined with confocal laser scanning microscopy (CLSM). Tensile properties were tested using an electronic universal testing machine (UTM) at 15 mm/min stretching rate. TG evaluated the thermal stability and RDS content in the fibers. A contact angle measuring instrument assessed the water wettability of each membrane.

To study the release profile of Si and Mg ions and membrane stability, samples (8 mg) were immersed in 4 ml of PBS (pH 7.4) and serum-supplemented α-MEM at 37^◦^C with gentle agitation. The eluates were collected at specific times, and Si and Mg ion concentrations were measured by ICP-OES. The membrane’s dry weight and the PBS solution’s pH were recorded pre- and post-soaking, and weight loss at predetermined time points was calculated. SEM was used to examine fiber morphology before and after degradation.

All core-shell membranes were immersed at a mass-to-volume ratio of 8 mg/4 ml in centrifuge tubes containing 4 ml of simulated body fluid (2×SBF), and incubated in a 37°C constant temperature shaking incubator, with medium changes every 2 days. On days 4, 7 and 14, the fibrous membranes were taken out, cleaned and dried. SEM and EDS were used to evaluate the formation of apatite on the surface of the fibrous membranes, assessing the scaffolds’ *in vitro* mineralization capability.

### In vitro cell experiments

Before cell culture, all samples were cut into 14 mm discs and sterilized using 15 kGy *γ*-ray irradiation. Due to the membranes’ adsorption of assay agents, leaching media was prepared by soaking them in α-MEM containing 10% NBCS and 1% Pen Strep for 48 h. The viability, proliferation and cytotoxicity of BMSCs on the membranes were evaluated using CCK-8 and Live/Dead Viability Kit assays. After 3 days, the adhesion of the BMSCs cultured on the membranes was observed via SEM following rinsing, fixation, dehydration and drying of the membranes. The cytoskeletons of BMSCs on the membranes were visualized using CLSM after staining with Hoechst 33342 and Alexa Fluor 488. After culturing on the membranes for 7 days, ALP activity was measured with an ALP Assay Kit. After 21 days of culture, calcium mineralization was determined by alizarin red S (ARS) staining. Osteogenic gene expression (ALP, COL1, OPN) in BMSCs after 7 days on the membranes was analyzed using RT-PCR, with primers listed in Supplementary Table S3. After 7 days of culture with the membranes, we analyzed the expression of the OPN protein in BMSCs using western blot (WB). Subsequently, we conducted a statistical analysis of the results using ImageJ software.

### In vivo experiments

All animal experiments in this study adhered to the Guide for the Care and Use of Laboratory Animals and received approval from West China Hospital Experimental Animal Ethics Committee (20230918004). Male Sprague Dawley rats (260–280 g) were randomly divided into three groups (sham, P-PG, 10RP-PG) for assessing subcutaneous implantation responses and the membranes’ effects on critical-sized calvarial bone defect repair. All samples were sterilized prior to implantation.

### Subcutaneous implantation

P-PG and 10RP-PG fibrous membrane discs (6-mm diameter) were implanted into the rats’ dorsal subcutaneous pockets. The sham group underwent surgery without membrane implantation. Tissue samples were collected at 3, 7 and 14 days post-surgery, paraffin-embedded, sectioned and stained with hematoxylin and eosin (H&E) and CD31 immunohistochemistry.

### Critical-sized calvarial bone defect repair

Two 5 mm diameter cylindrical defects were created on each side of the calvarial bone. In the experimental group, a sterilized fibrous membrane (8 mm diameter) covered each defect completely, while the sham group underwent surgery without implantation. Rats were euthanized at 4 and 8 weeks post-operation, and their skulls were harvested and fixed. Neo-bone formation was analyzed using micro-CT at 55 kV and 135 µA. The samples were then decalcified with EDTA, paraffin-sectioned and stained with H&E, Masson, ALP, OPN, COL1 and TRAP. To semi-quantify the immunohistochemistry staining, we conducted a statistical analysis using Image Pro Plus.

### Transcriptome sequencing analysis

Two weeks post-implantation in calvarial bone defects, skull samples from each group (*n* = 3) were collected. RNA sequencing was conducted using Illumina NovaSeq 6000 at Majorbio Biopharm Technology Co., Ltd, Shanghai. Differentially expressed genes (DEGs) were identified using DESeq2 software, considering a fold change >2 and a *P* values <0.05. Gene Ontology (GO) enrichment, Kyoto Encyclopedia of Genes and Genomes (KEGG) pathway analysis, and Gene Set Enrichment Analysis (GSEA) were performed on the Majorbio Cloud Platform.

### Statistical analysis

Data are presented as means ± SD. Statistical differences between samples were determined using one-way ANOVA followed by Tukey’s post-test in SPSS, with significance set at *P* < 0.05.

## Results and discussion

### Optimization of preparation method

**Figure 1.**
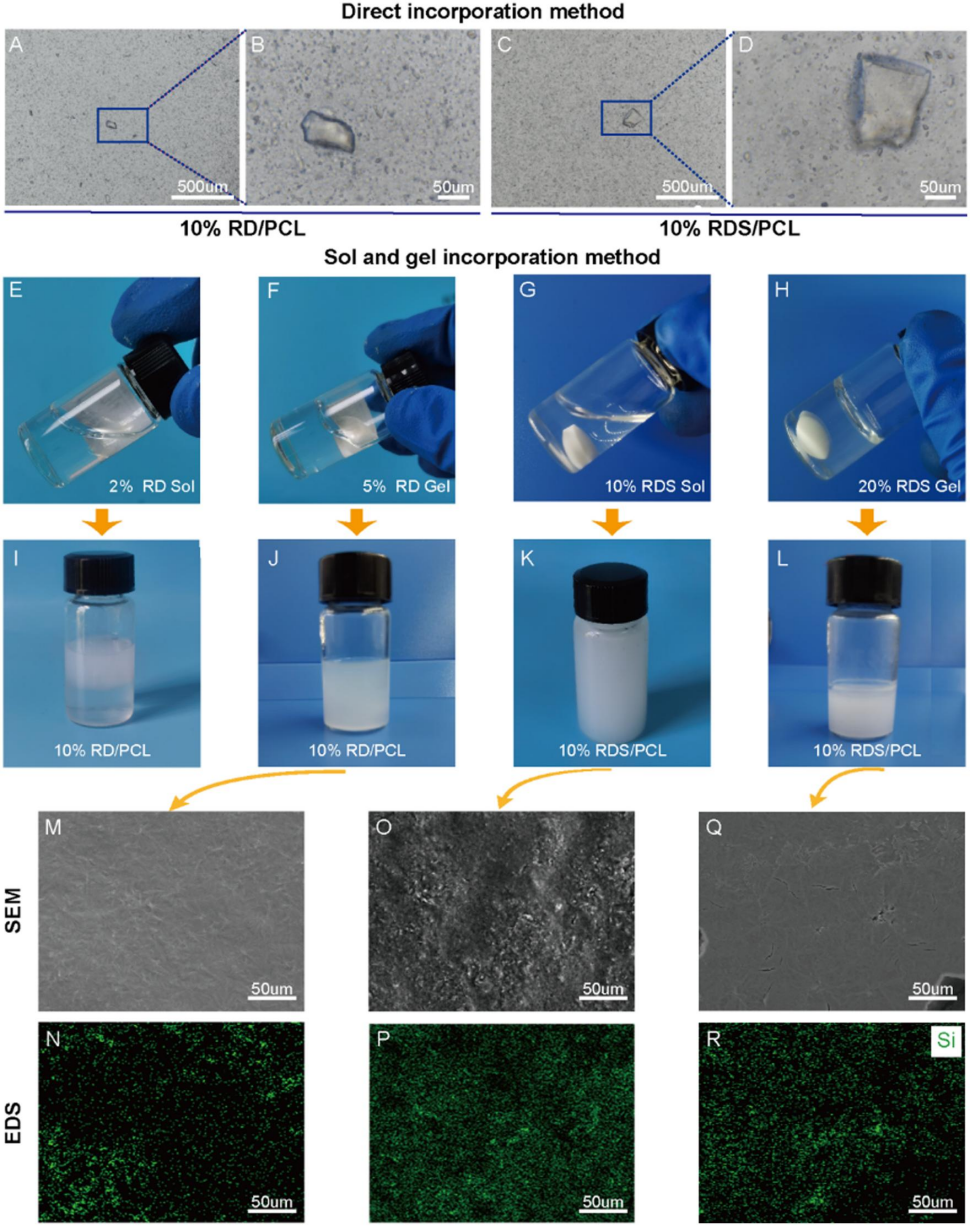
Inverted microscope images of the 10% RD/PCL (**A**, **B**) and 10% RDS/PCL (**C**, **D**) solutions prepared by the direct incorporation method. Images of laponite aqueous dispersions (**E**–**H**). images of 10% RD/PCL (**I**, **J**) and 10% RDS/PCL (**K**, **L**) solutions prepared by the aqueous dispersions incorporation method. SEM (**M**, **O**, **Q**) and EDS mapping (**N**, **P**, **R**) images of films prepared by 10% RD/PCL and 10% RDS/PCL solutions.

Gel-grade laponite (RD) and sol-grade laponite (RDS) were separately added to PCL solutions and stirred magnetically for 24 h, resulting in 10% RD/PCL (RD: PCL = 10:90, w/w) and 10% RDS/PCL (RDS: PCL = 10:90, w/w) solutions. However, inverted microscope observations showed that neither gel-grade nor sol-grade laponite achieved uniform dispersion in the PCL solution (Figure 1A–D).

Laponite easily disperses in water under agitation. Therefore, this study initially prepared laponite aqueous dispersion using RD and RDS. The results showed that 2% RD solutions (RD: DIW = 2:100, w/v) formed stable sols after 24 h of magnetic stirring (Figure 1E), while 5% RD solutions (RD: DIW = 5:100, w/v) quickly gelled upon dispersion in DIW (Figure 1F). For RDS, 20% concentrations (RDS: DIW = 20:100, w/v) quickly formed gels in DIW (Figure 1H), but 10% RDS solutions (RDS: DIW = 10:100, w/v) remained stable sols after 24 h of stirring (Figure 1G). This is because RDS contains the surface modifier tetrasodium pyrophosphate (Na_4_P_2_O_7_), which will prevent the formation of thixotropic gel structures [22].

Then, the obtained laponite aqueous dispersions were dispersed into PCL solutions, respectively to create 10% RD/PCL and 10% RDS/PCL solutions. It was observed that the 10% RD/PCL solution prepared with 2% RD sol could not mix uniformly due to phase separation (Figure 1I), and large RD particles were visible in the film of 10% RD/PCL solution made with 5% RD gel (Figure 1N). In contrast, fine RDS particles were homogeneously distributed in the film of 10% RDS/PCL solutions prepared with 10% RDS sol (Figure 1P). However, the uniformity of RDS particle dispersion in 10% RDS/PCL solutions prepared with 20% RDS gel was inferior to that prepared with 10% RDS sol (Figure 1R). Hence, 10% RDS sol was chosen to prepare various concentrations of RDS/PCL solutions for subsequent experiments.

**Figure 2.**
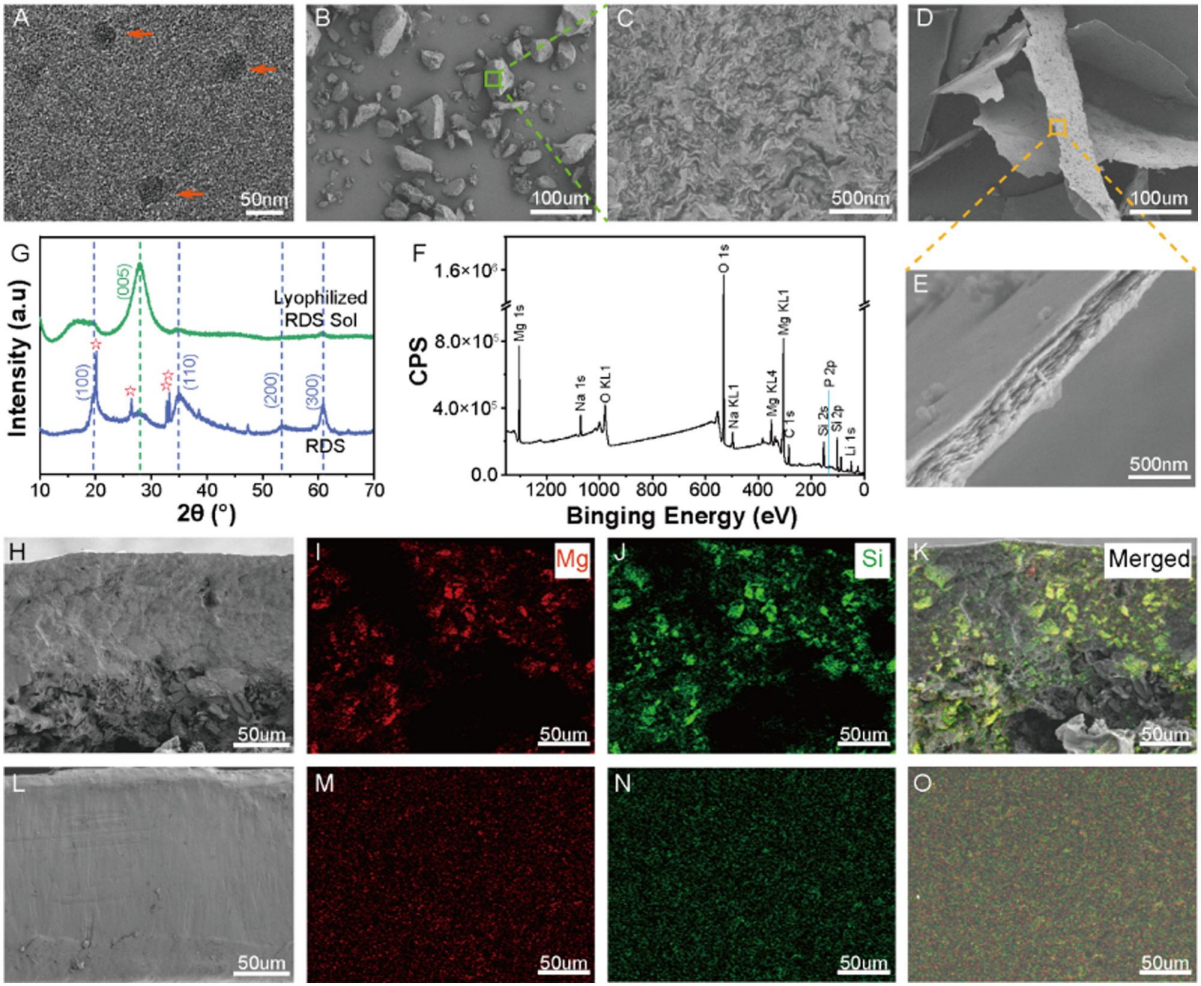
(**A**) TEM image of dispersed RDS powder. Arrows: laponite nanoparticles. (**B**, **C**) SEM images of RDS powder and (**D**, **E**) freeze-dried RDS sol. (**F**) XPS pattern of RDS powder. (**G**) XRD pattern of RDS powder and freeze-dried RDS sol. Stars: Na_4_P_2_O_7_. SEM images and EDS mappings of the cross-section of the RDS/PCL films prepared by direct (**H**–**K**) and sol incorporation methods (**L**–**O**).

The physical and chemical properties of RDS were detected first. TEM analysis showed that individual RDS nanoparticles took on a disc shape (20–50 nm) (Figure 2A). SEM images displayed the RDS powder as block-like, with a surface of scattered, stacked disc-shaped nanoparticles (Figure 2B and C). After freeze-drying of RDS sol, the disk-shaped RDS nanoparticles arranged themselves into layered, lamellar structures (Figure 2D) with a thickness of around 367 nm (Figure 2E). The results showed that the dispersion of RDS in water enabled the disaggregation of disk-like nanoparticles from bulk RDS, forming a sol. Subsequent freeze-drying led to the formation of organized, layered structures of RDS nanoparticles. XPS analysis of the freeze-dried RDS sol confirmed the presence of primary elements Si, Mg, O, Na (from laponite) and Na, P (from tetrasodium pyrophosphate), matching the theoretical composition (Figure 2F). EDS mapping further validated the XPS analysis results (Supplementary Figure S1).

XRD analyses revealed characteristic RDS diffraction planes (Figure 2G). Sharp RDS diffraction peaks (red stars) ascribed to Na_4_P_2_O_7_ were identified at 2θ of 20.15°, 26.47°, 32.76° and 33.23° [23]. The diffraction peaks for RDS powder, including those at 2θ  =  19.52°, 34.94°, 53.51° and 60.89° correspond to (100), (110), (200) and (300) crystal planes, respectively, were indicative of 2:1 layered octahedral montmorillonite-type clay minerals [24]. Meanwhile, the characteristic diffraction peak of the (005) crystal plane attributed to layered silicate clay also appeared at 2θ  =  27.94°. Freeze-drying RDS sol led to diminished (h00) and enhanced (005) plane intensities, indicating improved crystalline order, and corroborating SEM observations.

RDS/PCL solutions of varying concentrations (10%, 20%, 30% and 40%) were prepared using the 10% RDS sol incorporation method and freeze-dried to form stable RDS/PCL composites. The distribution of RDS within these composites, after redissolving and film formation, was analyzed using SEM and EDS mapping. The results demonstrated that compared to 10% RDS/PCL films prepared by the direct incorporation method, which featured by non-uniform distribution of large laponite particles (Figure 2H–K), those prepared by the sol incorporation method showed a finer and more uniform laponite distribution (Figure 2L–O). Meanwhile, 10% RDS/PCL composites developed by sol incorporation method were free from the cracking observed in films made by direct incorporation, indicating superior chemical stability in the sol-incorporated composites [25]. Similarly, RDS particles in 20%, 30% and 40% RDS/PCL films, prepared by adding 10% RDS sol to PCL solutions, also showed uniform dispersion (Supplementary Figure S2). Moreover, our extended experimental results demonstrated that RDS sol can be uniformly distributed in high-viscosity solutions, such as PU and CS (Supplementary Figure S2). These results suggest that laponite sol doping is a versatile method for creating composites with high filler content and uniform dispersion.

To assess the RDS/PCL composite’s spinnability, uniaxial electrospun fibrous membranes (5RP, 10RP, 20RP, RDS: PCL = 5:95, 10:90, 20:80, w/w) were prepared and analyzed. Results confirmed the suitability of RDS/PCL composite for electrospinning, with the successful incorporation of RDS into 10RP nanofibers (Supplementary Figure S3). To optimize the shell-spinning solution, Gel/PLGA fibrous membranes with varying gelatin contents were produced and evaluated. It was found that higher gelatin content led to more filamentous fibers but reduced the membranes’ elastic modulus and tensile strength (Supplementary Figure S4). Therefore, a PLGA solution with 10% gelatin (10PG, gelatin: PLGA = 10:100) was chosen as the optimal formulation to fabricate the shell of the coaxial nanofibers.

To study the shape stability of the fibrous membranes with varied compositions and structures, coaxial nanofibers (10RP-PG, 20RP-PG, 30RP-PG, core: 10RP, 20RP, 30RP, shell: 10PG) with shell-core flow ratios of 1:1, 2:1 and 3:1, and a uniaxial nanofiber (10PG) were fabricated. The anti-shrinkage properties of these membranes were assessed through an immersion test (Supplementary Figure S5A and B). At last, considering the influence of core-shell fibrous membrane composition and shell-core flow ratio on anti-shrinkage performance, we opted for a shell-core flow ratio of 2:1 and an RDS concentration of not more than 20% to fabricate coaxial nanofiber membranes for subsequent studies.

### Morphology and coaxial structure of fibers

**Figure 3.**
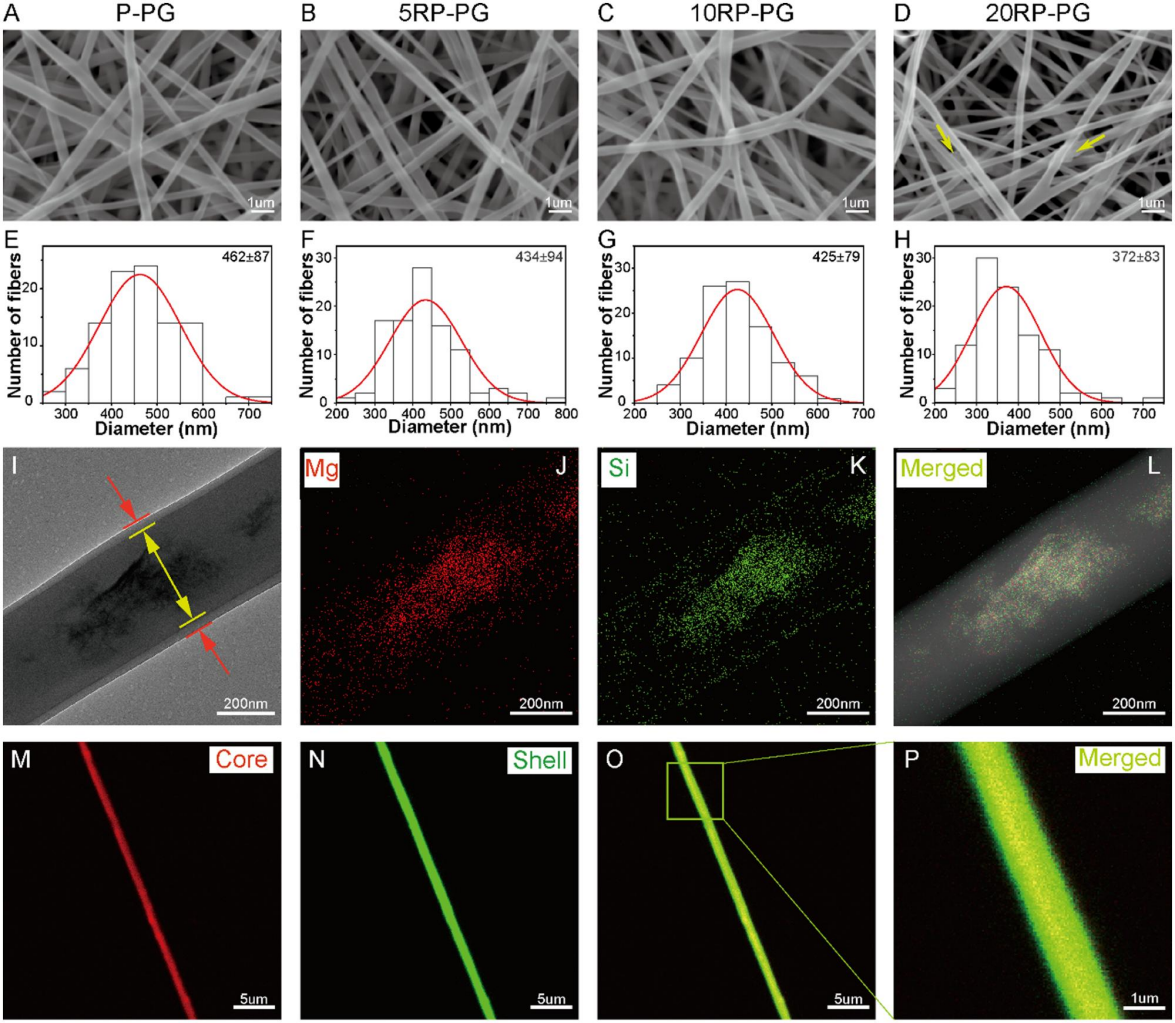
SEM images of (**A**) P-PG, (**B**) 5RP-PG, (**C**) 10RP-PG, (**D**) 20RP-PG electrospun nanofiber and their corresponding diameter distribution (**E**–**H**). (**I**) TEM image, (**J**–**L**) EDS mapping and (**M**–**P**) CLSM images of 10RP-PG core-shell structure fiber.

SEM images revealed that the fibers in P-PG, 5RP-PG, 10RP-PG and 20RP-PG had a random arrangement, mimicking natural ECM (Figure 3A–D), with fiber diameter decreasing as RDS content increased (Figure 3E–H). The 20RP-PG group had significantly smaller fiber diameters than the other groups (*P* < 0.01), and the 10RP-PG group’s fibers were also significantly thinner than those of the P-PG group (*P* < 0.05). TEM confirmed the core-shell structure in 10RP-PG fibers (Figure 3I), while EDX analysis verified Mg and Si presence, indicating uniform RDS distribution (Figure 3J–L). CLSM images revealed red and green dyed core and shell fibers, respectively, with yellow hues in the overlap area, further confirming the core-shell structure (Figure 3M–P). These results collectively validated the successful engineering of the core-shell fiber structure.

### Physical and chemical properties of membranes

Optimal strength is essential for guided bone tissue regeneration membranes to ensure effective suturing and mechanical support. Mechanical tests revealed that tensile strength and elastic modulus of electrospun membranes decreased with higher RDS content in the core (Supplementary Figure S5C–E). However, all membranes’ Young’s modulus fell within the characteristic range of cancellous bone (20–500 MPa) [26], suggesting the membranes can withstand tissue-imposed stresses and provide a conducive structural and mechanical environment for tissue regeneration.

**Figure 4.**
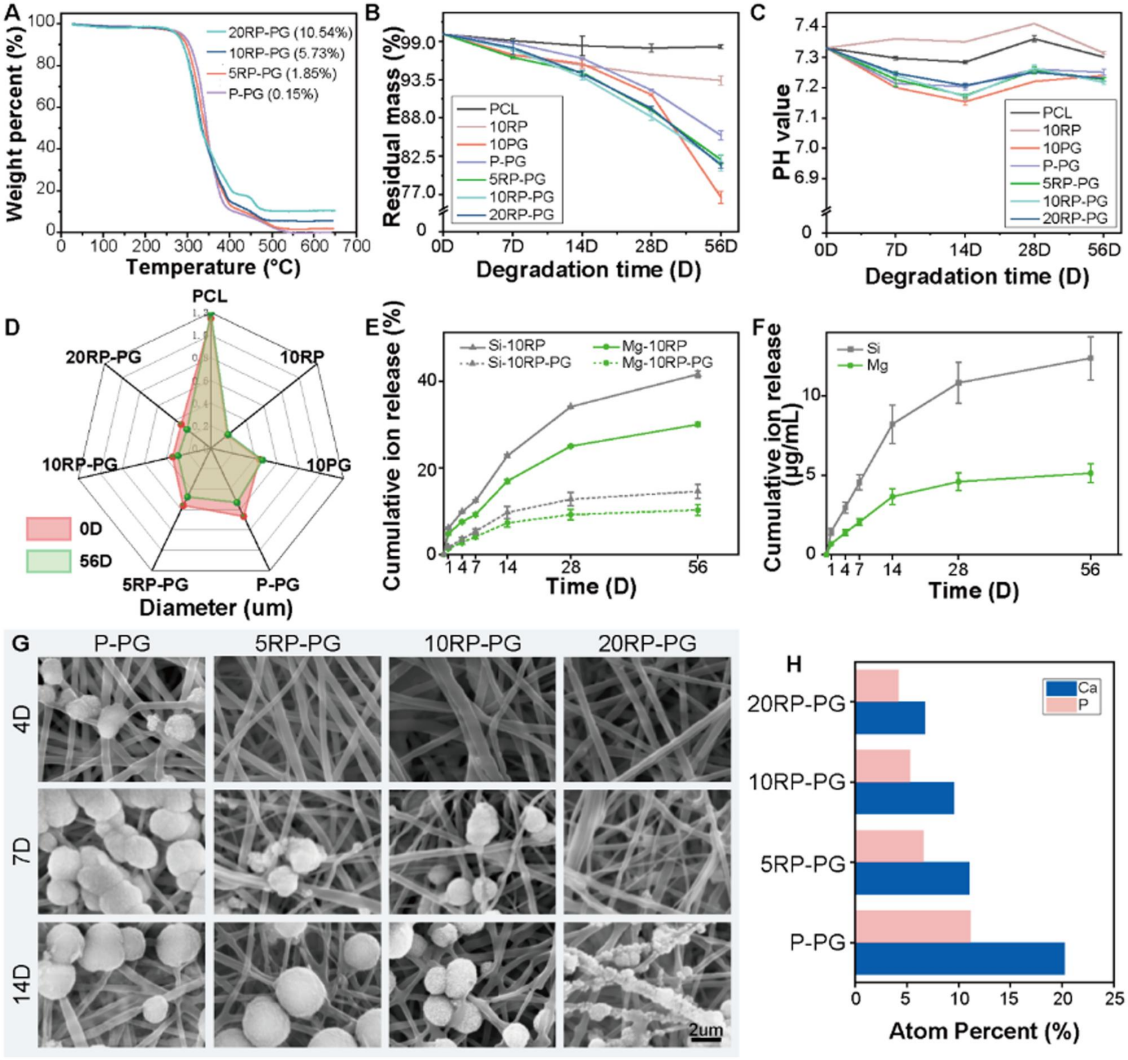
(**A**) TG curves. (**B**) Residual mass of degradation membranes. (**C**) PH value of degradation solution. (**D**) Fiber diameters pre- and post-56 days degradation. (**E**) The release behavior of Si and Mg from the 10RP and 10RP-PG membranes. (**F**) Si and Mg ion release curves of 10RP-PG membranes. (**G**) SEM images of fibrous membranes after 4, 7 and 14 days of mineralization in 2× SBF solution. (**H**) EDS analysis of the atom percentage of calcium and phosphorus in the mineralized fibrous membrane after 14 days of mineralization.

The thermogravimetric test revealed RDS concentrations of approximately 1.85 wt%, 5.73 wt% and 10.54 wt% in 5RP-PG, 10RP-PG and 20RP-PG membranes, respectively (Figure 4A). The data also reveal that the amount of RDS added had an insignificant effect on the thermal decomposition behavior of the membranes.

The degradation behavior PCL, 10RP and 10PG uniaxial fibers, as well as P-PG, 5RP-PG, 10RP-PG and 20RP-PG coaxial fibers, are depicted in Figure 4B. Among these, PCL fibrous membranes exhibited the slowest degradation rate. The incorporation of RDS resulted in an increase of degradation rate in 10RP group. The 10PG group showed the fastest degradation rate. Coaxial P-PG fibrous membranes had a moderate degradation rate. The introduction of RDS in the core layer marginally increased the degradation rate compared to P-PG, but the degradation rates among 5RP-PG, 10RP-PG and 20RP-PG were statistically indistinguishable.

Figure 4C illustrates the pH trends during degradation. The 10PG group showed a notable pH decrease, while the PCL group maintained a stable higher pH throughout the degradation process. The pH of 10RP exceeded that of the PCL group. Core-shell fiber groups’ pH levels were higher than 10PG’s but significantly lower than PCL’s. The difference in pH trends is attributed to PLGA’s rapid degradation and acidic by-products, in contrast to PCL’s slow degradation, which maintains structural integrity for over 56 days (Supplementary Figure S6). Additionally, the increased pH can be attributed to the mild alkalinity of RDS, and its exfoliation and dissolution from the fibers.

Morphological observation of degraded fibers (Supplementary Figure S6) showed that PCL and 10RP fiber surfaces remained largely unchanged over the degradation period. From Day 14 onwards, pores began to appear on the surface of 10PG, P-PG, 5RP-PG, 10RP-PG and 20RP-PG fibers due to the rapid degradation of gelatin, facilitating the release of RDS from the core in the coaxial fibrous membranes. In addition, we used 10RP as a representative of uniaxial and 10RP-PG of coaxial fibrous membranes to examine their degradation in the α-MEM medium compared to PBS, observing no significant differences (Supplementary Figure S7A). Fiber diameter analyses before and after 56 days of degradation (Figure 4D) showed that PCL and 10RP fibers underwent slight diameter increases, likely due to minor swelling of PCL after immersion. Despite later-stage degradation, initial shrinkage in the 10PG fibrous membrane resulted in thicker 10PG fibers compared to pre-immersion. As the PCL in the core layer mitigated membrane shrinkage, the diameter of all coaxial fibers decreased with the degradation of 10PG in the shell layer.

### In vitro release behavior of Si and Mg ions

Figure 4E–F illustrates the cumulative release profiles of Si and Mg ions from 10RP uniaxial and the 10RP-PG core-shell fibrous membrane in a neutral buffer solution. Throughout the release period, ions from all the membranes exhibited an initial rapid release followed by a slower release rate. Notably, the release rate from core-shell fibers was substantially slower than that from the uniaxial fibers. The release of Si ions from 10RP uniaxial and 10RP-PG core-shell fibrous membranes in α-MEM medium further confirmed the core-shell structure’s ability to provide a sustained slow release of bioactive components from the core (Supplementary Figure S7B). This suggests that 10RP-PG core-shell fibers enable sustained release, facilitating the long-term release of RDS dissolution and degradation products.

By Day 56, the Si ion concentration from the 10RP-PG core-shell membrane reached 12.40 µg/ml, falling within the effective osteogenesis-stimulating range of 10–50 µg/ml and well below its toxic level of 230 µg/ml [27–30]. The released Mg ion concentration measured 5.14 µg/ml, near the effective range of 6–12 µg/ml for osteogenesis and significantly below its toxic threshold of 240 µg/ml [31, 32]. A previous study noted that maintaining low Mg ion level in the early stage of osteogenesis can ensure normal collagen production of cells, supplying the essential raw materials for mineralization. A persistently high concentration of Mg ions would interfere with calcium ion positioning around collagen, altering the size and shape of hydroxyapatite (HA) and leading to suboptimal mineralization [31]. The stable and sustained release of Mg ions at later stage is crucial for guiding bone-related cells toward osteogenic differentiation and finally supporting bone reconstruction [33].

### In vitro mineralization

We further accessed the effect of RDS on the *in vitro* mineralization capacity by immersing the RDS-loaded fibrous membranes in 2× SBF solutions for 4, 7 and 14 days (Figure 4G and H). The results revealed that after 4 days, there was a slight mineral deposition on the surface of the P-PG fibrous membrane, while no mineral deposition was observed on the surfaces of the membranes loaded with RDS. After 7 days, a large amount of mineral deposition was observed on the surface of the P-PG fibrous membrane, whereas only a small amount of mineral deposition was observed on the surfaces of the 5RP-PG and 10RP-PG fibrous membranes, and no mineral deposition was observed on the surface of the 20RP-PG fibrous membrane. After 14 days, mineral deposition was observed on the surface of all the membranes. However, the quantity of mineral deposition was inversely proportional to the RDS content. This inverse relationship can be attributed to the presence of a small amount of tetrasodium pyrophosphate in RDS. Tetrasodium pyrophosphate is an effective inhibitor of crystal nucleation and growth, directly impeding the formation of hydroxyapatite [34–36]. Consequently, early-stage mineralization of the RDS-loaded membrane is significantly inhibited, while with the SBF being replaced every other day, the content of pyrophosphate continues to decrease, reducing its impact on the later mineralization of the fibrous membrane.

Based on morphological, mechanical and *in vitro* mineralization results, we selected the 5RP-PG and 10RP-PG groups to explore their *in vitro* cytocompatibility and osteogenic induction activity.

### Cell culture

**Figure 5.**
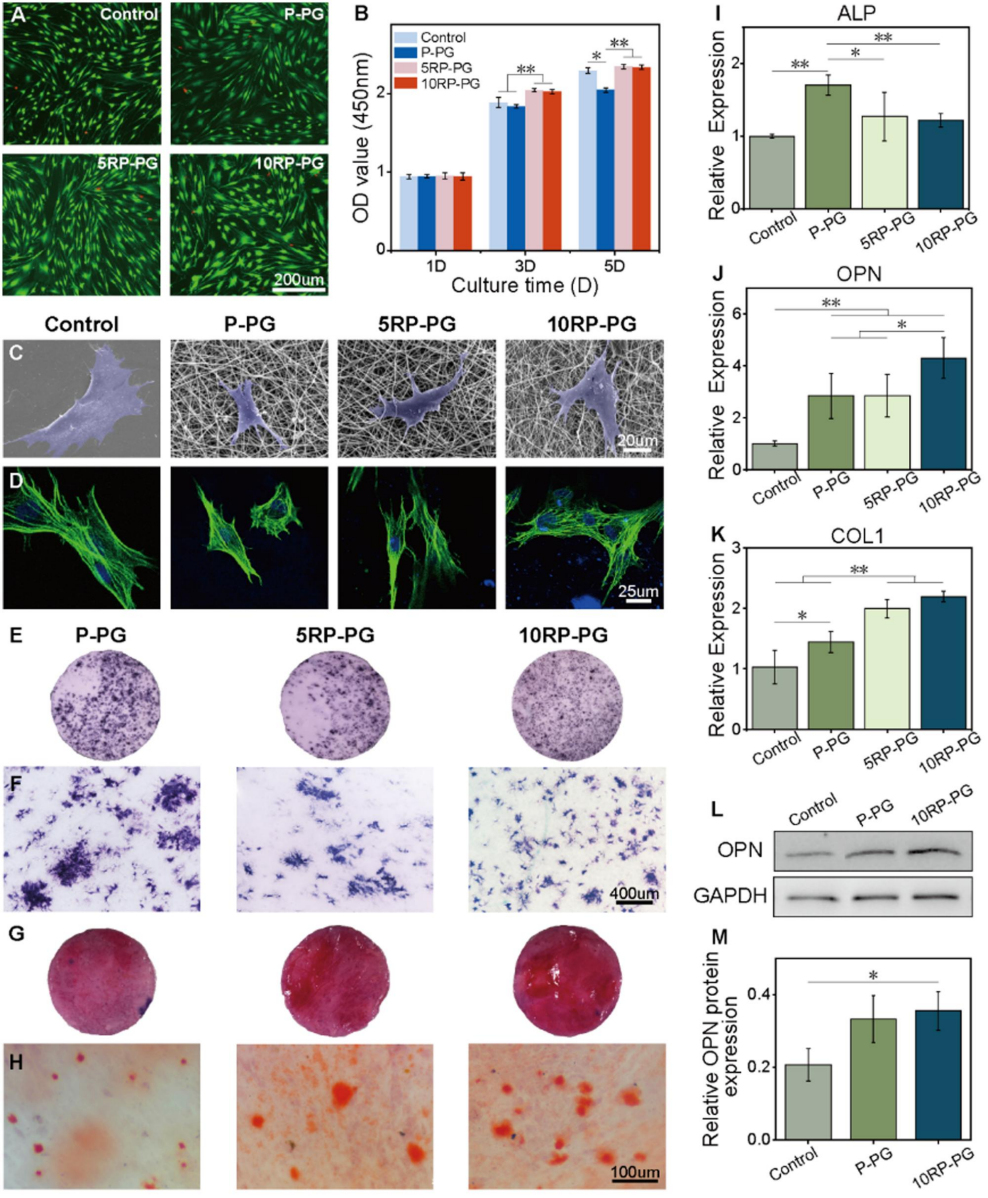
Cytocompatibility and osteogenic activity of core-shell membranes. (**A**) Live/dead assay showing cell viability after 3 days of culturing in the extracting liquid of different core-shell membranes. (**B**) Cell proliferation activity of BMSCs cultured with the extracting liquid of membranes. SEM (**C**) and CLSM (**D**) images of BMSCs cultured on fibrous membranes after 3 days. ALP staining images (**E**) and their magnified view (**F**) of BMSCs cultured on membranes for 7 days. ARS staining images (**G**) and their magnified view (**H**) of mineralization formation by BMSCs cultured on membranes for 21 days. Relative expression of (**I**) ALP, (**J**) OPN and (**K**) COL1 genes in BMSCs cultured on fibrous membranes after 7 days. WB analysis (**L**) and its quantification (**M**) of the expression of OPN in BMSCs cultured on fibrous membranes for 7 days. *P < 0.05 and **P < 0.01.

After a 3-day culture in the leaching liquid of various fibrous membranes, live/dead staining showed that the majority of BMSCs displayed high viability, as indicated by green fluorescence, while a minimal number of cells showed signs of death (red fluorescence) (Figure 5A). The CCK-8 test showed that BMSCs cultured in the extracts of all test groups proliferated over the culture period (Figure 5B). At Day 3, both 5RP-PG and 10RP-PG groups displayed higher proliferation activity compared to the control and P-PG groups. By Day 5, the P-PG group showed the lowest rate of proliferation. To observe cell–material interactions, BMSCs were cultured on membranes for 3 days and subsequently observed by SEM and CLSM. SEM images showed that BMSCs cultured on RDS-loaded membranes were more flattened and covered a larger area than those on the P-PG membrane (Figure 5C). After being stained with F-actin, CLSM showed that BMSCs could successfully adhere to both tissue culture plates and the fibrous membranes (Figure 5D). The cell nuclei appeared normal, and an abundance of actin microfilaments was observed in all groups. Notably, cells attached to the P-PG fiber membrane were spindle-shaped and exhibited limited spreading, while cells on the 5RP-PG and 10RP-PG fibrous membranes showed extensive spreading with clear and interconnected filopodia—favorable for intercellular communication [37]. These results indicate that the addition of an appropriate amount of RDS enhances both cell adhesion and growth.

To investigate osteogenesis induced by the membranes, we cultured BMSCs on the membranes. We then evaluated alkaline phosphatase (ALP) activity in BMSCs with ALP staining at 7 days post-culture, assessed mineralized nodule formation with ARS staining at 21 days post-culture, and analyzed gene expression associated with osteogenic differentiation at 7 days post-culture. ALP staining showed a decrease in ALP activity with increasing RDS content in the membranes (Figure 5E and F). Conversely, ARS staining demonstrated a higher presence of mineralized nodules on the 10RP-PG membranes than on the P-PG and 5RP-PG membranes (Figure 5G and H). PCR analysis indicated that RDS-loaded fibrous membranes downregulated ALP expression and upregulated osteopontin (OPN) and type I collagen (COL1), relative to P-PG (Figure 5I–K). Based on the ARS and PCR results, we selected the control, P-PG and 10RP-PG groups for WB analysis to determine the OPN protein expression, and the results were consistent with the PCR trends (Figure 5L and M).

The upregulating of COL1 gene expression may be related to the release of Si ions, which have been found to induce the synthesis of extracellular matrix proteins, such as COL1 [38, 39]. The decrease in ALP activity, along with the downregulation of ALP gene expression and the upregulation of both OPN gene and protein expression, may be attributed to the pyrophosphate ions present in RDS. As inhibitors of HA formation, pyrophosphate ions inhibit the mineralization of osteoblasts by binding to minerals, inhibiting ALP activity and upregulating OPN [40]. However, as substrates for phosphatase enzymes, pyrophosphate may be cleaved by the osteoblast-related enzyme (ALP) [41]. *In vitro* evidence suggested that supplementing of exogenous enzyme accelerated the dissolution of the inorganic pyrophosphate ions, causing a simultaneous complete loss of their mineralization inhibition and a localized elevation in orthophosphate ion concentration [40]. Therefore, over the 21-day cell culture period, regular medium exchanges removed some pyrophosphate, and the ALP synthesized by the cells decomposed the remaining pyrophosphate. This process reduced pyrophosphate’s inhibitory effect on mineralization. Simultaneously, the decomposition of pyrophosphate generated phosphate, which is beneficial for mineralization [42]. Additionally, laponite significantly induces osteogenic differentiation in BMSCs [43, 44]. Consequently, the 10RP-PG group, which contains a higher concentration of laponite, has produced a larger quantity of calcified nodules. Moreover, *in vivo* study showed that the presence of the pyrophosphate in the material did not inhibit the mineralization of the healing bone around the implant, but actually appeared to stimulate it [45]. These findings mean that the pyrophosphate ions, inhibiting apatite formation *in vitro*, may not inhibit bone formation *in vivo*, due to the abundance of ALP *in vivo*. Therefore, *in vivo* experimental evaluation was focused on the 10RP-PG group.

### In vivo evaluation

Firstly, we performed *in vivo* experiments to assess the biocompatibility of the fabricated membranes by subcutaneous implantation. The results confirmed the excellent biocompatibility and minimal foreign body reaction of 10RP-PG fibrous membranes (Supplementary Figure S8). We further conducted HE staining on the non-defective area (at the sagittal suture) of the critical-sized cranial defect model to study the degradation and biocompatibility of the fibrous membranes *in vivo*. The results showed that the fibrous membrane remained visible throughout the 8-week experimental period, with no distinct fibrous capsule formation around the material observed at both 4 and 8 weeks (Supplementary Figure S9). Moreover, a small amount of new bone formation was observed near the 10RP-PG fibrous membrane in the non-defective areas of the skull. This suggests that the 10RP-PG fibrous membrane has excellent biocompatibility and osteogenic activity.

**Figure 6.**
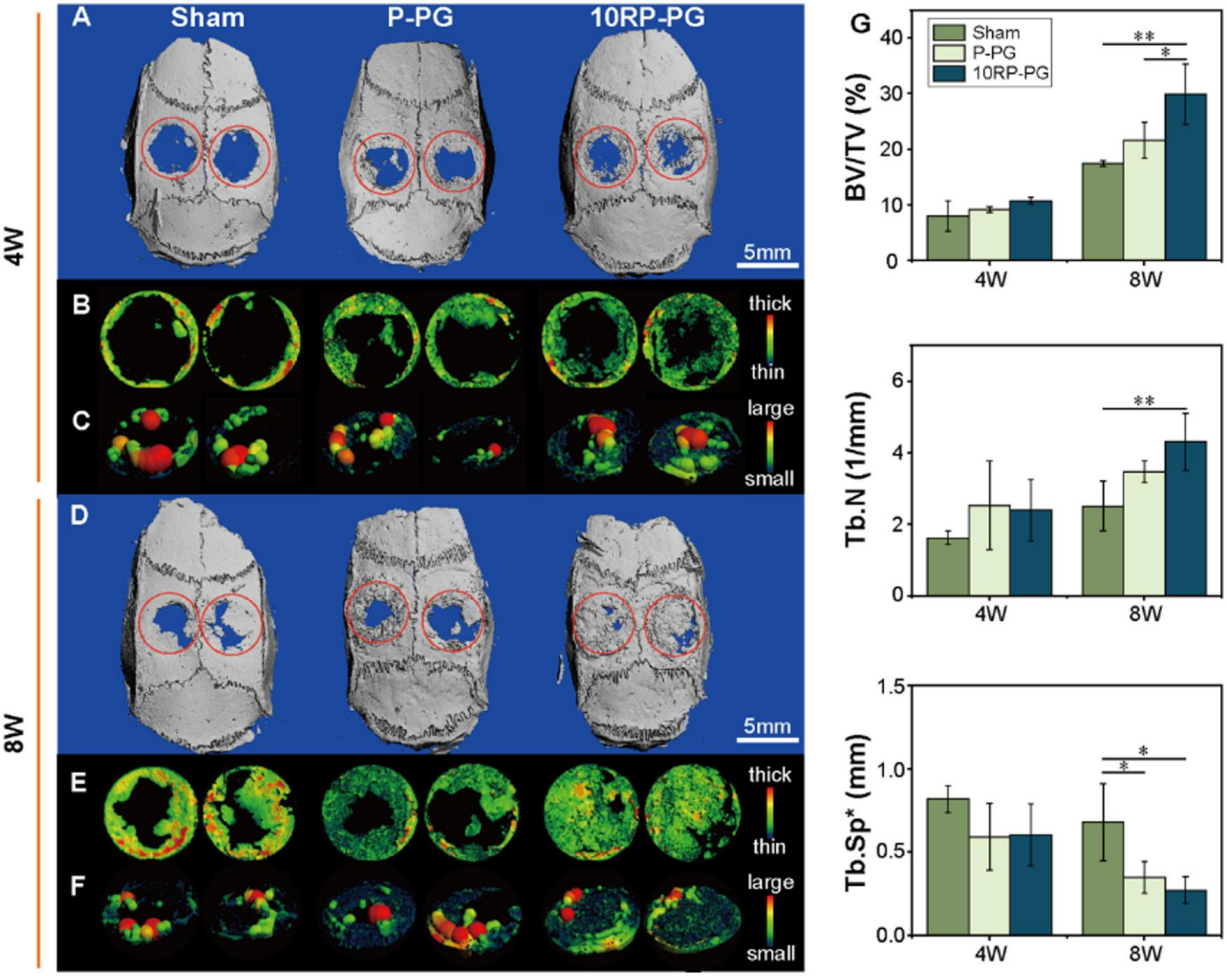
Micro-CT analysis at 4 weeks: (**A**) μ-CT panoramic view of the rat calvarial bone; images of neo-bone and trabecular thickness (Tb.Th) distribution (**B**) and Tb.Sp (**C**) in the defect area. (**D**–**F**) The corresponding Micro-CT analysis at 8 weeks. (**G**) Quantitative analysis of BV/TV, Tb.N and Tb.Sp. *P < 0.05 and **P < 0.01.

Subsequently, we assessed the *in vivo* osteogenic potential of P-PG and 10RP-PG fibrous membranes using the critical-sized calvarial bone defect model. The volume fraction of newly formed bone and the spatial structure of trabeculae were analyzed using micro-CT scanning after 4 and 8 weeks of implantation (Figure 6). At week 4, all groups exhibited minor new bone formation (Figure 6A). Quantitative analysis (Figure 6G) showed a hierarchy in terms of bone volume fraction (BV/TV): 10RP-PG > P-PG > Sham, although the difference between P-PG and 10RP-PG groups was not statistically significant. After 8 weeks, new bone almost completely covered the defect area in the 10RP-PG group, confirming its superior osteogenic efficacy (Figure 6D). Further quantitative metrics (Figure 6G) supported this observation, demonstrating the highest bone volume fraction and trabecular number (Tb.N), along with the lowest trabecular separation (Tb.Sp) for 10RP-PG. This suggests that the 10RP-PG fibrous membrane has exceptional osteogenic potential, underlining RDS as a bioactive substance efficacious in bone regeneration.

**Figure 7.**
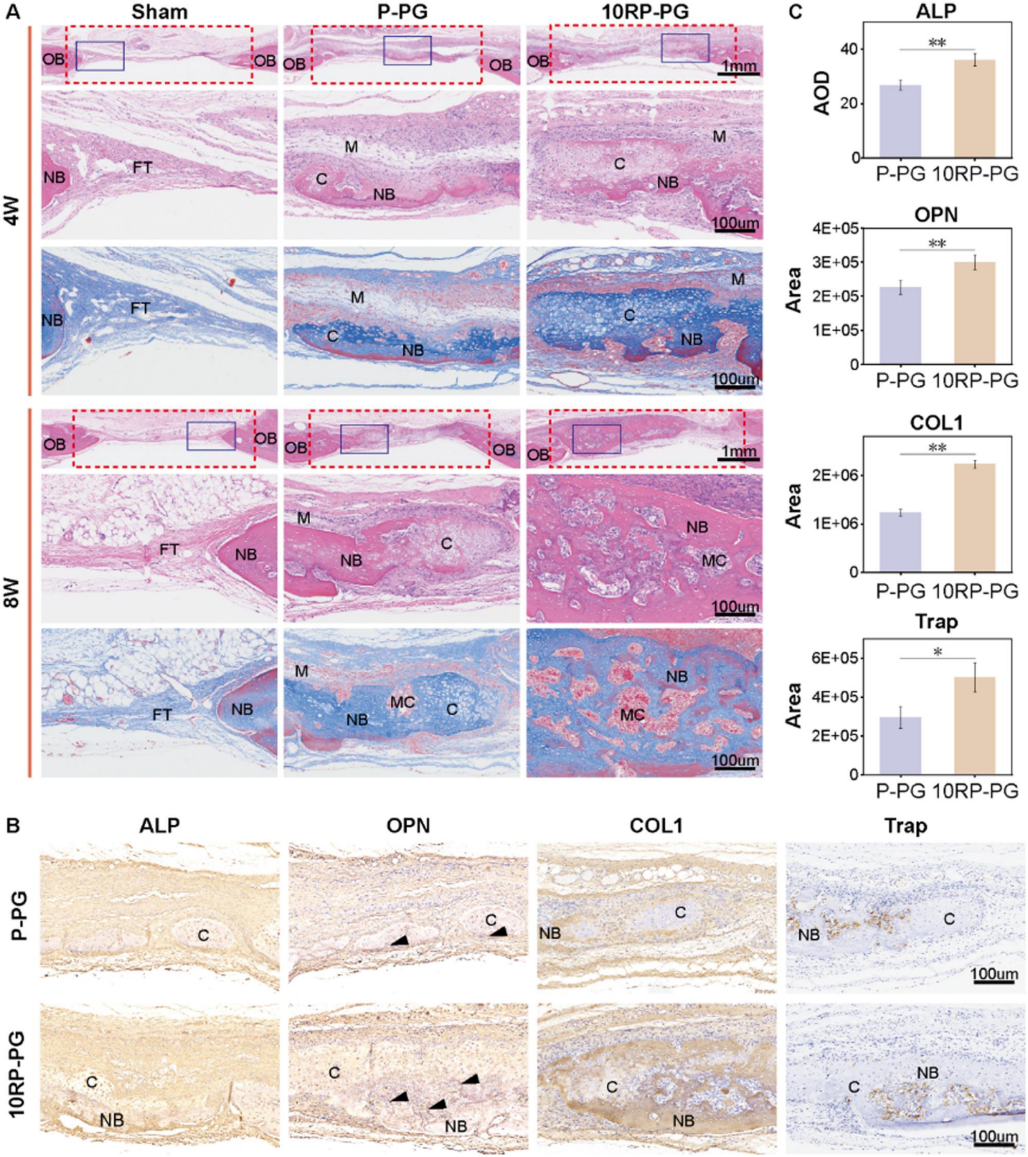
Histological section analysis. (**A**) H&E and Masson staining of the skull defect. Dashed lines mark the defect area. The solid-boxed regions represent the magnified areas below. (**B**) Immunohistochemical staining (ALP, OPN, COL1 and TRAP) of the rat calvarial defect at 4 weeks. The nuclei appear blue, and the positive expression is brownish-yellow. (**C**) Semiquantitative calculation of immunohistochemical staining. *P < 0.05 and **P < 0.01. FT, fibrous tissue; C, cartilage tissue; OB, old bone tissue; NB, new bone tissue; MC, marrow cavity; M, material; black triangle, OPN cement line.

Micro-CT findings were further confirmed by H&E and Masson staining (Figure 7A). At 4 weeks post-implantation, the defect area of the sham group was largely filled with fibrous connective tissue, and only minimal new bone formation was observed. Both cartilage and bone tissues were present in the bone defect areas in the P-PG and 10RP-PG groups, although the volume of new bone was limited. Notably, the 10RP-PG group displayed more cartilage tissue than the P-PG group. At 8 weeks, there was still only a small amount of new bone formation in the control group. In contrast, the P-PG group displayed an increased presence of both cartilage and bone tissues. Remarkably, the bone defect area in the 10RP-PG group was nearly completely filled with new bone tissue with a bone marrow cavity inside.

The results substantiate that bone repair in the material groups predominantly occurs via endochondral ossification [46, 47]. In comparison to P-PG, 10RP-PG expedited this process. At 4 weeks, increased cartilage formation was evident, and by 8 weeks, cartilage was almost completely resorbed, giving way to new bone tissue and entry into the bone remodeling stage.

The ability of membranes to promote osteogenesis at 4 weeks was assessed using immunohistochemistry (Figure 7B and C). ALP, a glycoprotein, can act as an exoenzyme attached to the outer surface of cells and matrix vesicles [48]. Stronger ALP expression is observed in the 10RP-PG group compared to the P-PG group in the sections. It has been reported that ALP activity is associated with initial mineralization [49, 50]. ALP promotes extracellular mineralization by hydrolyzing the mineralization inhibitor inorganic pyrophosphate to release inorganic phosphate [51]. OPN secretion appears to be one of the earliest matrix secretion events by (pre-) osteoblasts during the bone remodeling cycle. The secreted OPN accumulates on the resorbed bone surfaces, forming a cement line situated between the newly formed bone tissue and the original bone tissue. In our sections, the P-PG group exhibited only a small amount of OPN cement lines at the cartilage edges, whereas the 10RP-PG group showed a greater amount of OPN cement lines. This suggests that the 10RP-PG group experienced a higher degree of cartilage matrix resorption and greater formation of new bone tissue compared to the P-PG group. Additionally, being a highly charged and phosphorylated protein, OPN has a high affinity for calcium, rendering it a key player in the mineralization process of bone tissue. Type I collagen constitutes approximately 95% of the total collagen content in bone, yet it is not present in hyaline cartilage [52]. From the images, it is evident that the proportion of cartilage is higher than that of new bone in the P-PG group. Conversely, in the 10RP-PG group, the proportion of new bone is greater than that of cartilage. TRAP staining showed a higher level of positive expression in the 10RP-PG group compared to the P-PG group. Within the cartilage plate, TRAP-positive multinucleated cells are believed to play a significant role in the process whereby bone replaces the hypertrophic chondrocyte region [53].

The immunohistochemical results indicate that the 10RP-PG fibrous membrane, supplemented with RDS, effectively facilitated the expression of ALP and OPN, both associated with mineralization. Additionally, it markedly accelerated the process of endochondral ossification. Stronger TRAP activity led to the destruction of the cartilage plate more rapidly, promoting more new bone formation and the deposition of COL1, thereby ultimately achieving more efficient and effective bone regeneration.

### Transcriptomic insights into mechanisms of 10RP-PG mediated bone repair

**Figure 8.**
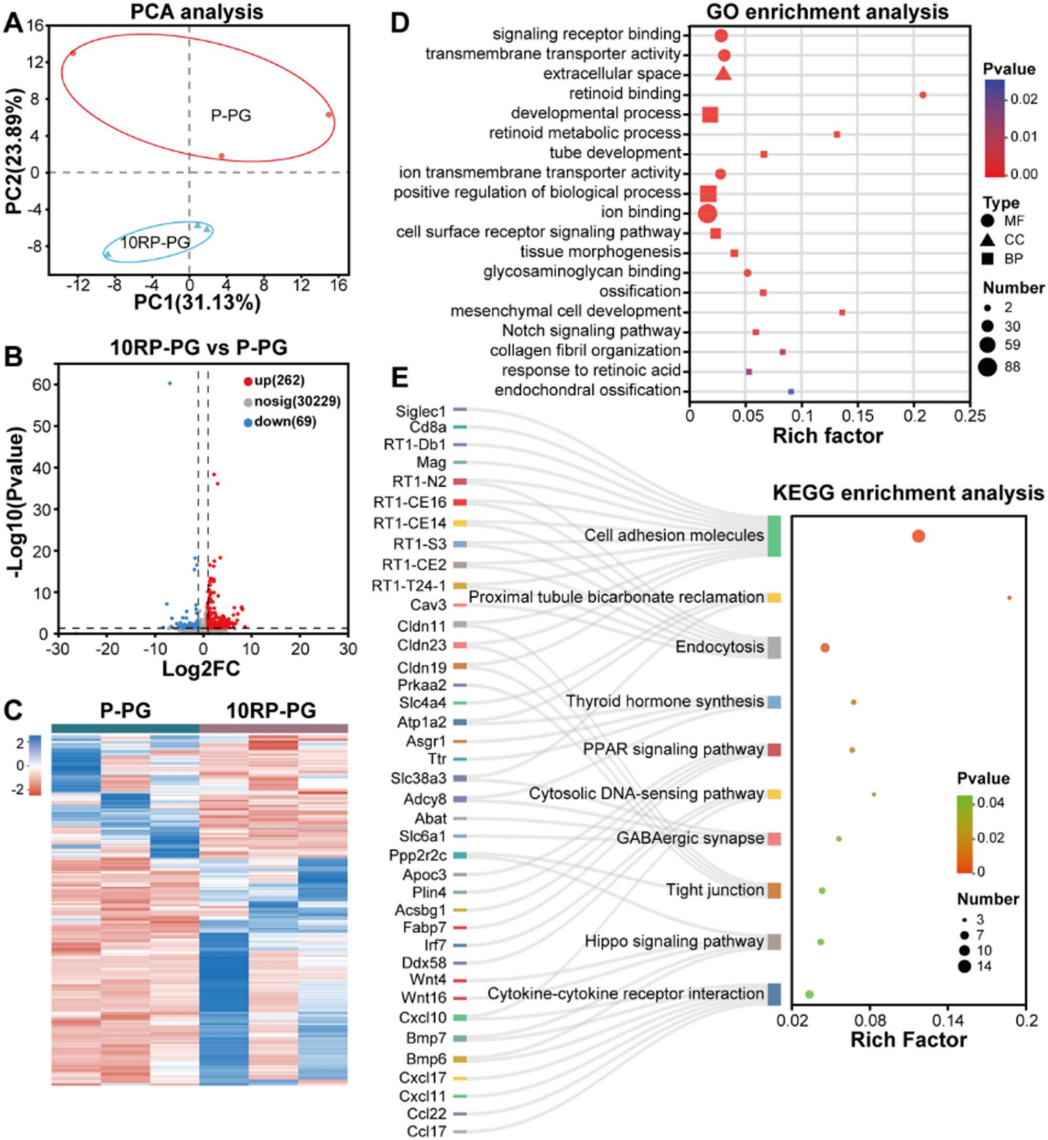
(**A**) PCA analysis between samples. (**B**) Volcano map and (**C**) heat map of DEGs. (**D**) GO analysis of DEGs and (**E**) KEGG pathway enrichment results.

To gain a deeper understanding of the underlying molecular mechanisms employed by 10RP-PG fibrous membranes in promoting bone repair, bulk RNA sequencing was conducted two weeks post-implantation. Principal component analysis (PCA) revealed significant transcriptomic differences between groups (Figure 8A), with 262 genes upregulated and 69 downregulated in the 10RP-PG group compared to P-PG (Figure 8B and C), indicating that the introduction of RDS caused significant differential gene expression.

Subsequently, GO enrichment analysis was applied to further determine the functions of these DEGs. Notably, DEGs are enriched in GO terms including signaling receptor binding (GO:0005102), transmembrane transporter activity (GO:0022857), ion transmembrane transporter activity (GO:0015075) and ion binding (GO:0043167). Further KEGG analysis indicated the activation of the endocytosis signaling pathway. It has been suggested that laponite particles are internalized into the cytoplasm through clathrin-mediated endocytosis and are subsequently degraded in lysosomes to release mineral ions such as Si, Mg and Li, which trigger biochemical signaling [24]. In the present study, KEGG and GO terms related to endocytosis and signaling were notably upregulated in the 10RP-PG group, supporting the notion that RDS undergoes cellular uptake via endocytosis, followed by the release of mineral ions that activate biochemical signaling critical for bone and cartilage formation.

Taking the GO terms as an example, activation of retinoid signaling (retinoid binding (GO:0005501), retinoid metabolic process (GO:0001523), response to retinoic acid (GO:0032526)) allow for a seamless transition from hypertrophic cartilage to endochondral bone [54]. Notch signaling (GO:0007219) regulates both endochondral and intramembranous bone healing and also plays a role in the regulation of angiogenesis, either directly or indirectly through receptors for vascular endothelial growth factor [55, 56]. Additionally, upregulated GO terms, including tube development (GO:0035295), mesenchymal cell development (GO:0014031), glycosaminoglycan binding (GO:0005539), collagen fibril organization (GO:0030199), ossification (GO:0001503), and endochondral ossification (GO:0001958), are beneficial for both endochondral ossification and ossification processes [57, 58].

Furthermore, KEGG signaling pathways related to osteogenesis, such as cell adhesion molecules (rno04514), Hippo (rno04390), Thyroid hormones synthesis (rno04918) and PPAR signaling pathway (rno03320) were also found to be activated (Figure 8E). Among them, bone cell adhesion to the extracellular matrix directly regulates cell growth, the expression of the osteoblast phenotype and the process of bone tissue formation [59]. Several members of the Hippo signaling pathway also play important roles in regulating osteoclast differentiation [60]. Thyroid hormones mediate endochondral ossification and linear growth [61]. Peroxisome proliferator-activated receptors work as heterodimers with retinoid X receptors (RXR), and the PPAR-RXR complex binds to specific responsive sequences on DNA resulting in target gene expression [62].

Meanwhile, GSEA was performed to refine our understanding of the functional roles of RDS in 10RP-PG membranes (Supplementary Figures S10 and S11). The results confirmed previous enrichment analyses (including clathrin-coated endocytic vesicle, chondrocyte development, osteoblast differentiation, bone growth, Hippo, Retinol metabolism, Notch) and identified additional pathways (such as canonical Wnt, Focal adhesion, Wnt and TGF-β signaling pathway) implicated in endochondral ossification and osteogenesis, further substantiating the multi-faceted roles of RDS in bone repair [63–65].

Considering the results of RNA sequencing, we hypothesize that RDS in 10RP-PG membranes is internalized by endocytosis. Its degradation products likely activate a range of signaling pathways crucial for endochondral ossification and osteogenesis. These pathways include, but are not limited to, Hippo, Notch, WNT and TGF-β. Collectively, the RNA-seq data corroborate our histomorphological results, affirming that 10RP-PG fibrous membrane supplemented with RDS can potentiate endochondral ossification and bone formation. This effect appears to be mediated through the release of ions that act as triggers for intracellular signaling pathways.

## Conclusion

In this study, we developed a core-shell fibrous membrane featuring a laponite-loaded PCL core layer and a gelatin/PLGA shell layer. Specifically, RDS sol was integrated into the PCL electrospinning solution, achieving a stable and homogeneous dispersion of laponite particles in the resultant fibers. The designed core-shell structure, coupled with the channels created by the rapid degradation of the gelatin in the outer layer, allowed for a controlled and prolonged release of bioactive ions (Si and Mg) over 56 days. Moreover, the obtained 10RP-PG core-shell fibrous membrane resolved the challenges of size instability associated with PLGA-based systems. Utilizing a critical-sized calvarial bone defect model, we observed that the 10RP-PG fibrous membrane significantly promoted endochondral bone formation. RNA-seq analysis further supported the histological findings by revealing the activation of multiple signaling pathways (Hippo, Notch, WNT and TGF-β) crucial for bone formation, as mediated by the release of bioactive ions from the RDS component. Taken together, our results indicate this core-shell fibrous membrane holds great promise for bone regeneration applications.

## Supplementary Material

rbae080_Supplementary_Data
