# Supplementary material for: Nano-laponite encapsulated coaxial fiber scaffold promotes endochondral osteogenesis
Source: Regen Biomater. 2024 Jul 4;11:rbae080. doi: 10.1093/rb/rbae080 (PMC11269679; doi:10.1093/rb/rbae080)
Supplement: rbae080_Supplementary_Data [file rbae080_supplementary_data.docx]

**Nano-laponite encapsulated coaxial fiber scaffold promotes endochondral osteogenesis**

Li Yuan^1^, Jiawei Wei^1^, Shiqi Xiao^1^, Shue Jin^1^, Xue Xia^1^, Huan Liu^1^, Jiangshan Liu^1^, Jiaxin Hu^1^, Yi Zuo^1^, Yubao Li^1^, Fang Yang^2,^* and Jidong Li^1,^*

*^1^Research Center for Nano-Biomaterials, Analytical and Testing Center, Sichuan University, Chengdu, 610064, PR China*

*^2^Department of Dentistry – Regenerative Biomaterials, Research Institute for Medical Innovation, Radboudumc, Nijmegen, The Netherlands*

*^*^Corresponding address. E-mail: fang.yang@radboudumc.nl (F. Yang); nic1979@scu.edu.cn (J. Li)*

**Table S1.** The materials used in the experiment and their manufacturers

| Material | Manufacturer |
| --- | --- |
| PLGA | Jinan Daigang Biomaterial Co., Ltd., China |
| PCL | Shenzhen Esun Industrial Co., Ltd , China |
| RD | BYK-Chemie GmbH . China |
| RDS | BYK-Chemie GmbH . China |
| Gelatin | Aladdin Co., Ltd, China |
| HFIP | Aladdin Co., Ltd, China |
| Rhodamine B | Beijing Solarbio Science & Technology Co., Ltd, China |
| Coumarin-6 | Aladdin Co., Ltd, China |
| α-MEM | Gibco, USA |
| NBCS | Gibco, USA |
| Pen Strep | MP Biomedicals, USA |
| CCK-8 | Meilunbio, China |
| Live/Dead Viability assay Kit | Life Technologies, USA |
| Hoechst 33342 | Life Technologies, USA |
| Alexa Fluor® 488 | Life Technologies, USA |
| ALP Assay Kit | Beyotime, China |
| Alizarin red solution | Soleibao Technology Co., LTD, China |
| Formalin-free Fixative | Jiangyuan^®^ |
| dexamethasone | Aladdin, Shanghai |
| β-sodium Glycerophosphate | Aladdin, Shanghai |
| Ascorbic Acid | Chengdu Kelong Chemical Reagent Co., Ltd. China |
| H&E reagent | Servicebio, China |
| CD31 antibody | Servicebio, China |
| Masson reagent | Servicebio, China |
| collagen Ⅰ antibody | Servicebio, China |
| TRAP antibody | Servicebio, China |
| OPN antibody | Servicebio, China |
| ALP antibody | Servicebio, China |

**Table S2.** Test instruments used in the experiment and their models

| Equipment | Model |
| --- | --- |
| TEM | JEM-F200, JEOL, Japan |
| SEM | Sigma 300, Zeiss, Germany |
| XRD | D8 Advance, Bruker, Germany |
| XPS | K-Alpha, Thermo Scientific, USA |
| FTIR | Nicolet 6700, USA |
| CLSM | AIR MP+, Nikon, Japan |
| UTM | AG-IC 50 KN, SHIMADZU, Japan |
| TG | STA499F3 Jupiter®, German |
| Contact angle measuring instrument | JY-82B, Chengde Dingsheng, China |
| ICP-OES | 5100 SVDV, Agilent, USA |
| Fluorescence Microscopy | Eclipse Ti-U, Nikon, Japan |
| Micro-CT | VivaCT80, SCANCO Medical AG, Switzerland |
| Light Microscope | TE 2000-U, Nikon Eclipse, Japan |
| Illumina NovaSeq 6000 | Illumina, USA |

**Table S3.** Nucleotide primers used for quantitative polymerase chain reaction

| Gene | Primers (5’ to 3’): Sense & Antisense |
| --- | --- |
| ALP | Forward: 5’-GCAGAAGCCGCCAACCTGTG-3’ |
|  | Reverse: 5’-CTGTCCTGAGCATCAGCATGAGTC3’ |
| OPN | Forward: 5’-AACACTCAGATGCTGTAGCCACTTG-3’ |
|  | Reverse: 5’-GCTTTCATTGGAGTTGCTTGGAAGAG-3’ |
| COL 1 | Forward: 5’-GCGAACAGGGCGACAGAGGCATAAAG-3’ |
|  | Reverse: 5’-GGACCAACAGGACCAGCATCACCAGT-3’ |
| GAPDH | Forward: 5’-CTGGAGAAACCTGCCAAGTATG-3’ |
|  | Reverse: 5’-GGTGGAAGAATGGGAGTTGCT-3’ |


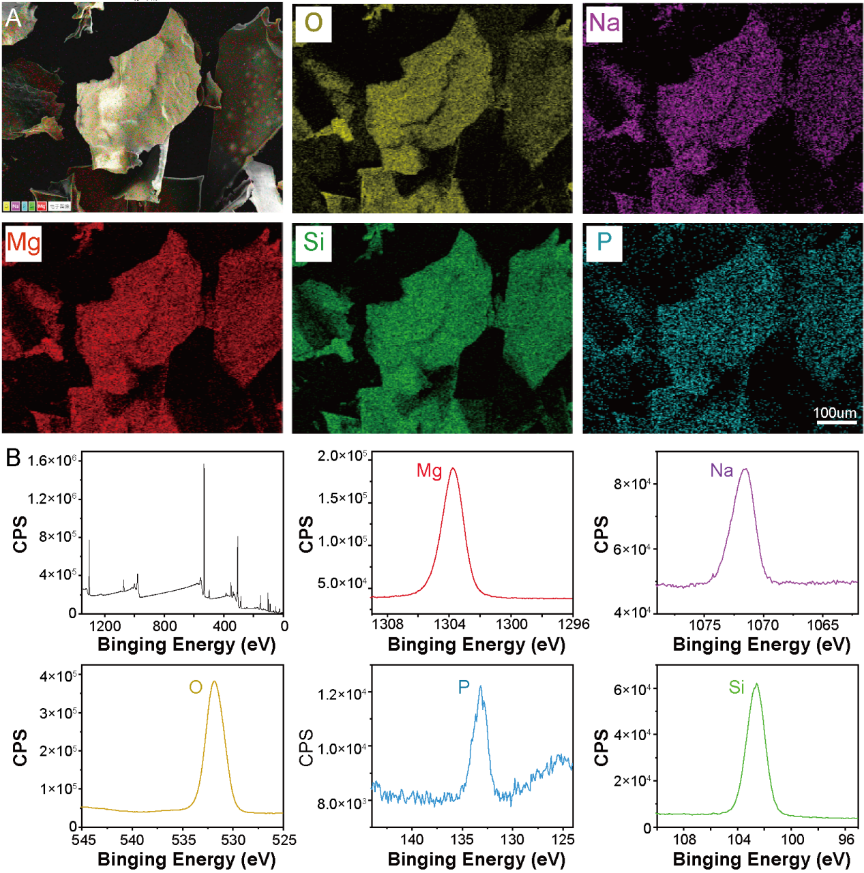


**Figure S1.** (A) Element distribution and EDS spectrum of RDS. (B) XPS spectrum of RDS.


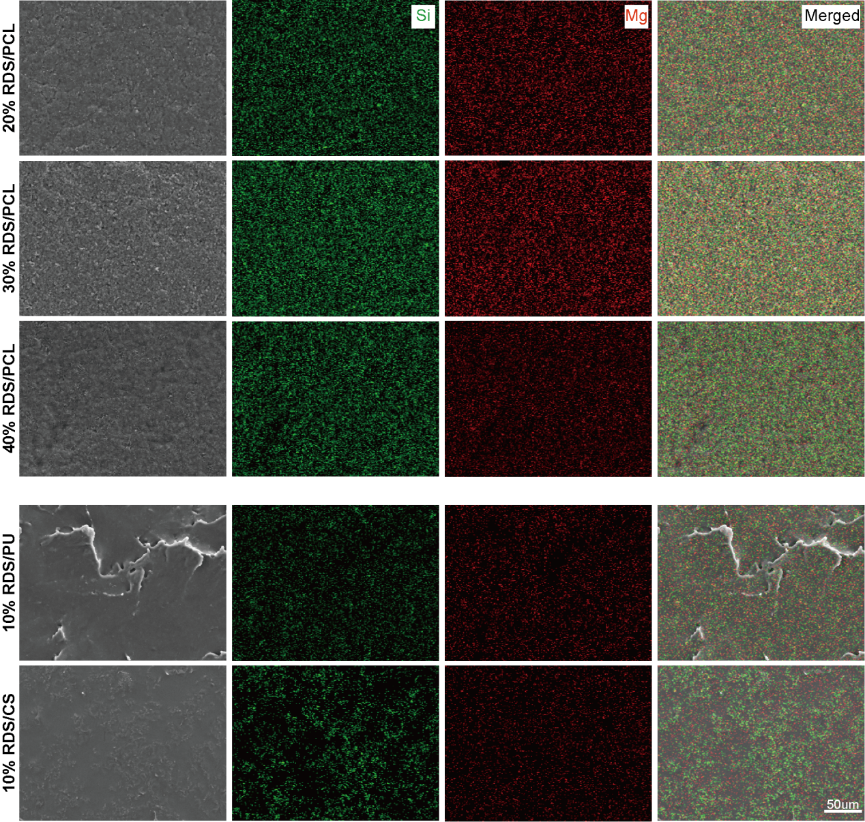


**Figure S2.** SEM and EDS mapping images of films prepared by 20% RDS/PCL, 30% RDS/PCL, 40% RDS/PCL, 10% RDS/PU, and 10% RDS/CS solutions.


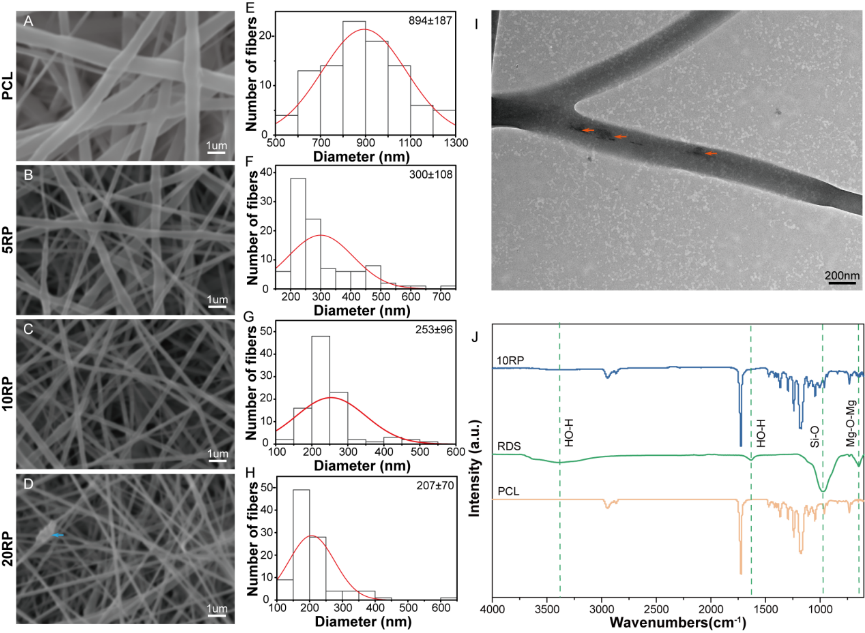


**Figure S3.** SEM images of (A) PCL, (B) 5RP, (C) 10RP, and (D) 20RP fibrous membrane. Blue arrows: beaded structure. (E-H) The corresponding diameter distribution of different fibrous membranes. (I) TEM image of 10RP fiber. Orange arrows: RDS. (J) FTIR spectra of RDS powder, PCL membrane, and 10RP membrane.

SEM images showed that the diameter of nanofibers decreased with the increase of the RDS concentration (Fig. S3A-H). This may be due to the enhanced electrical conductivity of the spinning solution following the addition of RDS, which in turn resulted in stronger fiber stretching during the electrospinning process. In addition, a small number of beaded structures can be observed in 20RP fibers. TEM demonstrated the successful incorporation of RDS into 10RP nanofibers (Fig. S3I).

FTIR spectral analysis of the membranes exhibited bands at 3385 cm^−1^, 1632 cm^−1^, 970 cm^−1^, and 646 cm^−1^, corresponding to the HO–H stretching vibrations, the bending HO–H bond of interlayer water in laponite, Si–O stretching vibrations, and Mg-O-Mg bending vibration, respectively (Fig. S3J) [1]. In the 10RP membrane, the HO-H stretching and bending were absent. Furthermore, the Si-O stretching vibration peak in the 10RP membrane overlapped partially with the PCL absorption peak at 961 cm^−1^, causing the Si-O peak to shift to 1000 cm^−1^. The Mg-O-Mg bending vibration peak was also detected in the 10RP membrane at 646 cm^−1^.


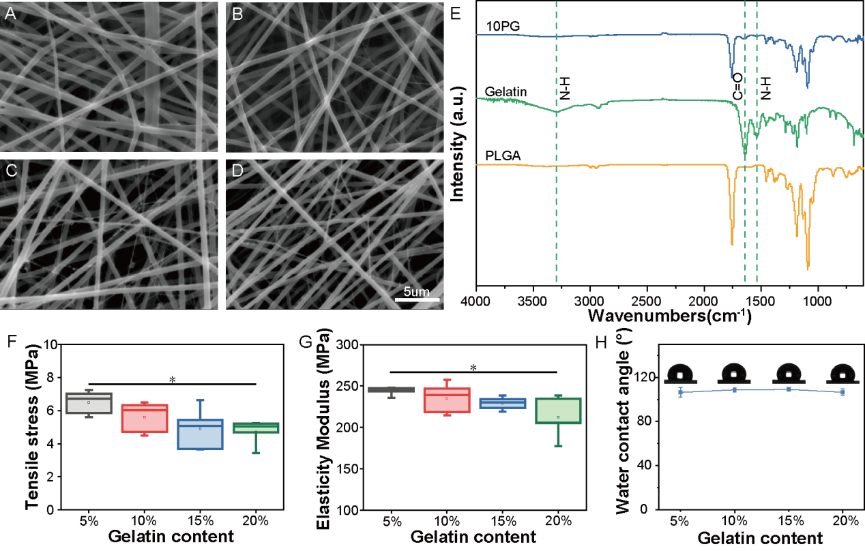


**Figure S4.** Optimization of shell spinning solutions. SEM images of PLGA/Gelatin membranes with ratio of Gelatin to PLGA (A) 5%, (B) 10%, (C) 15%, and (D) 20%. (E) FTIR spectra of PLGA, Gelatin, and 10PG membranes. (F) Tensile stress, (G) elasticity modulus and (H) water contact angle of fibrous membranes.

Increasing gelatin content led to more viscous spinning membranes that were increasingly difficult to peel off from the collector. SEM revealed that the presence of fine, filamentous fibers in the fibrous mat also increased with the gelatin content (Fig. S4A-D). Tensile tests showed that both the elastic modulus and tensile strength decreased as the gelatin content increased (Fig. S4F-G). The water contact angle remained relatively stable (Fig. S4H).

Spectral data from FTIR analysis showed peaks corresponding to different amide groups in gelatin and composite films. While the gelatin film exhibited prominent peaks at 3290 cm^−1^ (amide A), 1640cm^−1^ (amide I), and 1540 cm^−1^ (amide II), corresponding to NH stretching, CO stretching, and NH bending, respectively [2-4], the intensities of these regions decreased in the composite 10PG films (Fig. S4E). In addition, the peak corresponding to amide A was absent in the 10PG membranes.

**
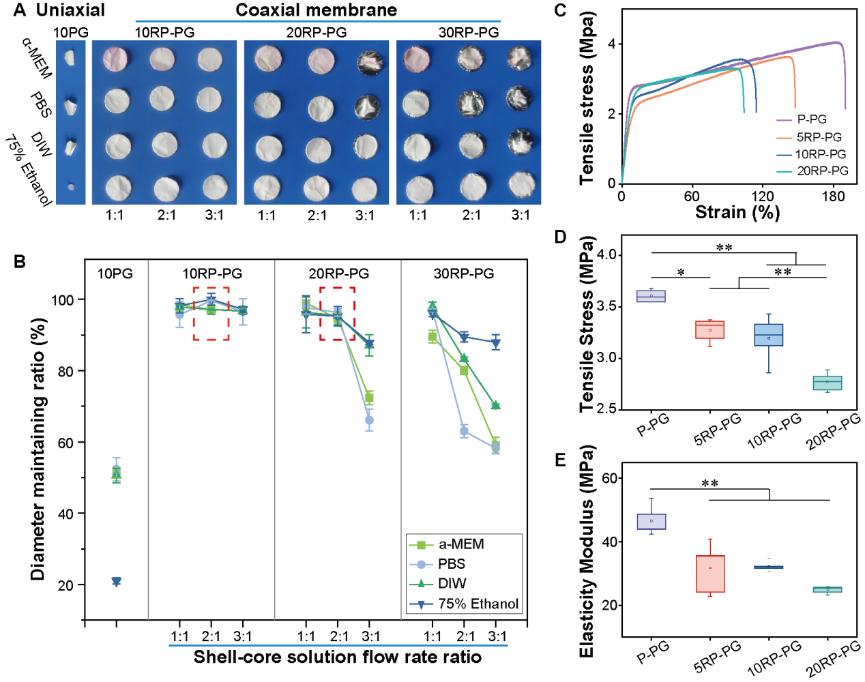
**

**Figure S5.** Characterization of anti-shrinkage properties of coaxial fibrous membranes: (A) The macroscopic photos of the fibrous membranes after immersion in liquid media for 24 h, (B) Corresponding quantitative statistical analysis of the diameter maintaining ratio. Mechanical properties of fibrous membranes: (C) stress-strain curves, (D) tensile stress, and (E) elasticity modulus.

It was evident that uniaxial 10PG nanofibers experienced significant shrinkage and curling after 24 H of immersion in liquid media (Fig. S5A-B). Conversely, adding RDS/PCL to the core layer enhanced the fibers’ resistance to shrinkage. Notably, the shell-core flow ratio also influenced the anti-shrinkage properties of the obtained membranes. Specifically, only 10RP-PG maintained its original shape at a 3:1 shell-core flow ratio. Both 10RP-PG and 20RP-PG nanofibers maintained their size and shape at a 2:1 ratio, and all coaxial nanofibers remained stable at a 1:1 ratio.

Given these findings, it is clear that an optimal flow rate ratio is crucial for preparing core-sheath nanofibers via coaxial electrospinning. Generally, the flow rate of the core solution is lower than that of the sheath solution [5]. Thereby, we opted for a shell-core flow ratio of 2:1 and RDS concentration of not exceeding 20% to fabricate nanofiber membranes for subsequent studies.


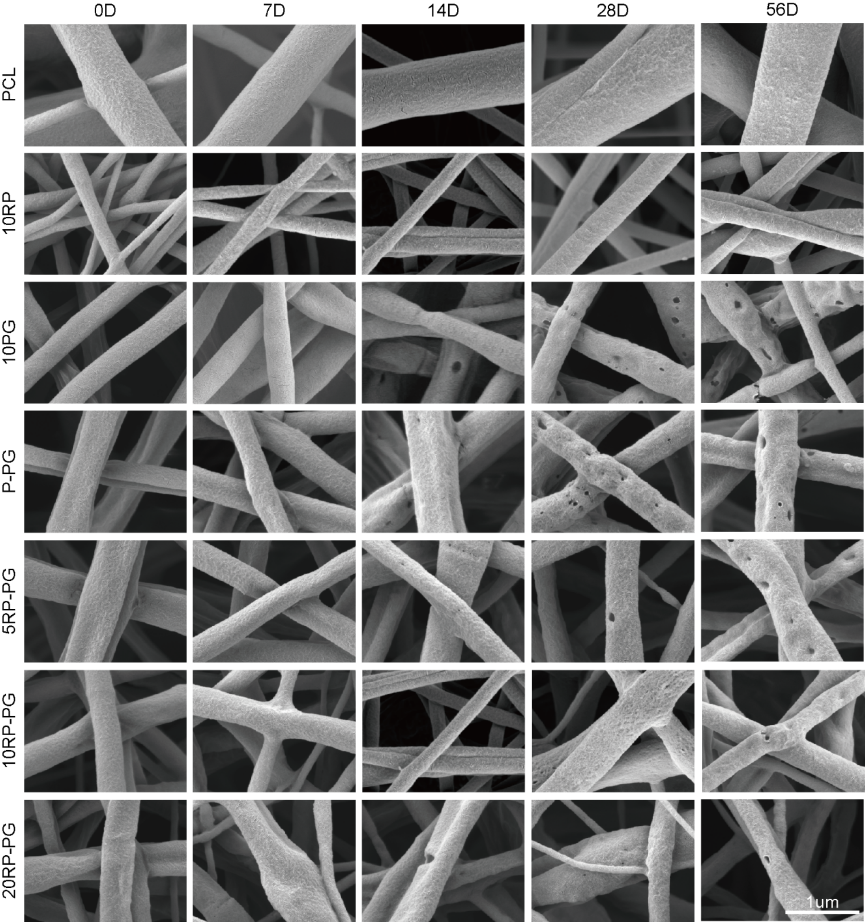


**Figure S6.** The SEM images of PCL, 10RP, 10PG, P-PG, 5RP-PG, 10RP-PG, and 20RP-PG fibrous membranes after degradation for 0, 7, 14, 28, and 56 days.


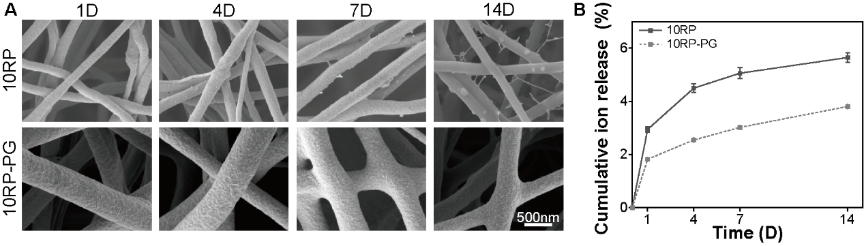


**Figure S7.** (A) The SEM images of 10RP and 10RP-PG fibrous membranes after degradation for 1, 4, 7, and 14 days in α-MEM medium. (B) The release behavior of Si from the 10RP and 10RP-PG membranes in α-MEM medium.

**
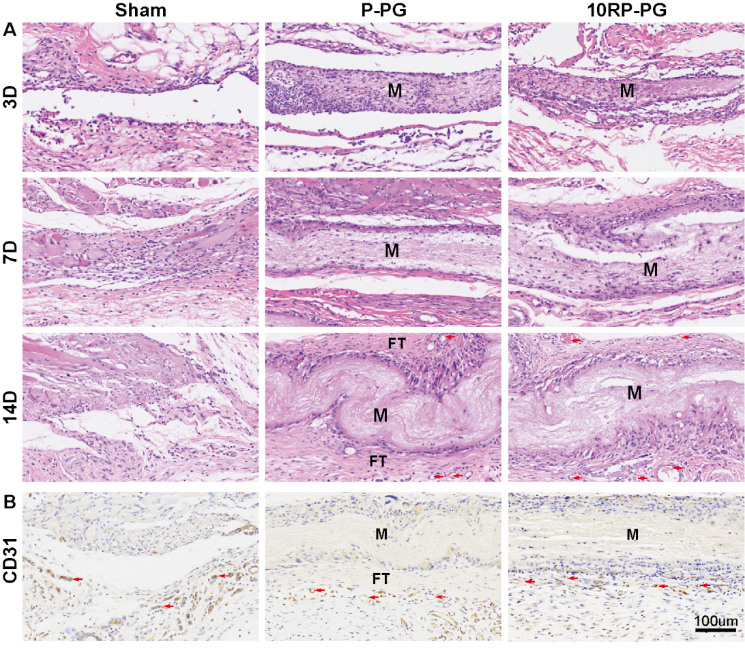
**

**Figure S****8.** (A) H&E staining of fibrous membranes after implanted subcutaneously in SD rats for 3, 7, and 14 days. (B) CD31 staining after 14 days of subcutaneous implantation of different fibrous membranes. M represents material, FT represents fibrous tissue, the red arrows indicate blood vessels.

Firstly, P-PG and 10RP-PG fibrous membranes were subcutaneously implanted into rats to assess host response. Three days post-implantation, a significant recruitment of immune cells was observed around both the fibrous membranes and the sham-operated site (Fig. S8A). Subsequently, inflammation around P-PG and 10RP-PG fibrous membranes gradually subsided at days 7 and 14 post-implantation, indicative of homeostatic regulation. CD31 staining of the 14-day samples revealed that a substantial blood vessel network around the 10RP-PG membranes (Fig. S8B), in contrast to the fibrous, but relatively avascular, tissue layer surrounding P-PG.


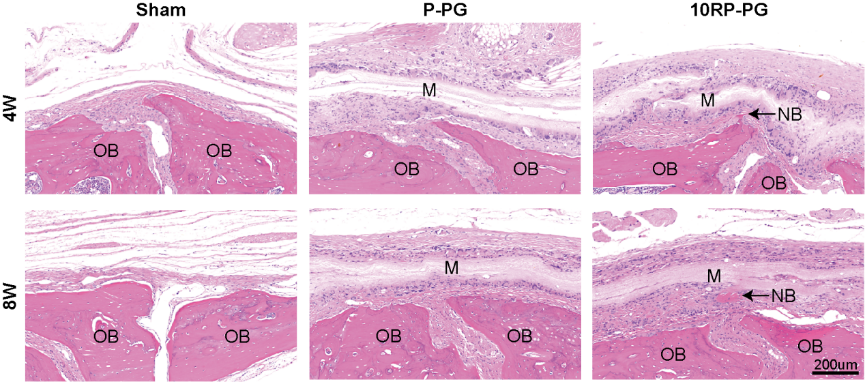


**Figure** **S9.** H&E staining images of the non-defect area (at the sagittal suture) of the critical-sized cranial defect model at 4 weeks and 8 weeks. OB, Old bone tissue; NB, New bone tissue; M, material.

**
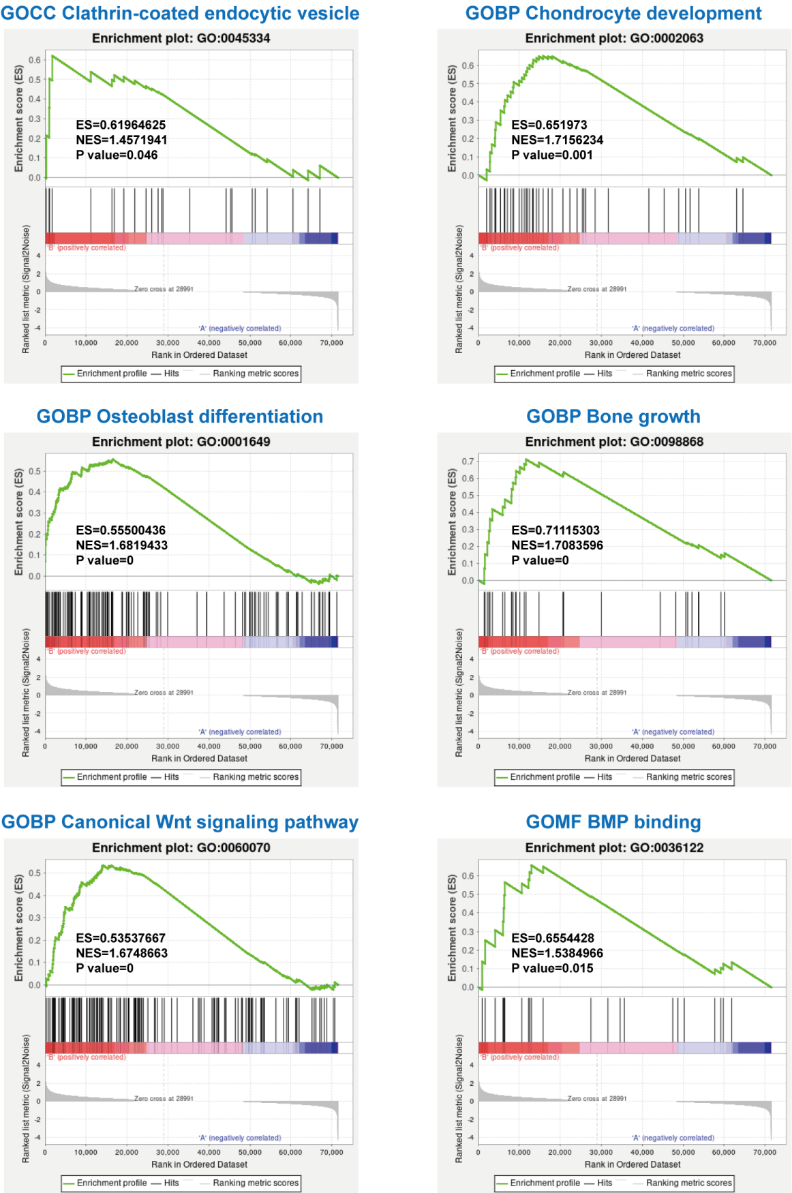
**

**Figure S10.** Significant GSEA plots (GO) associated with endocytosis, chondrocytes, and osteogenesis.

**
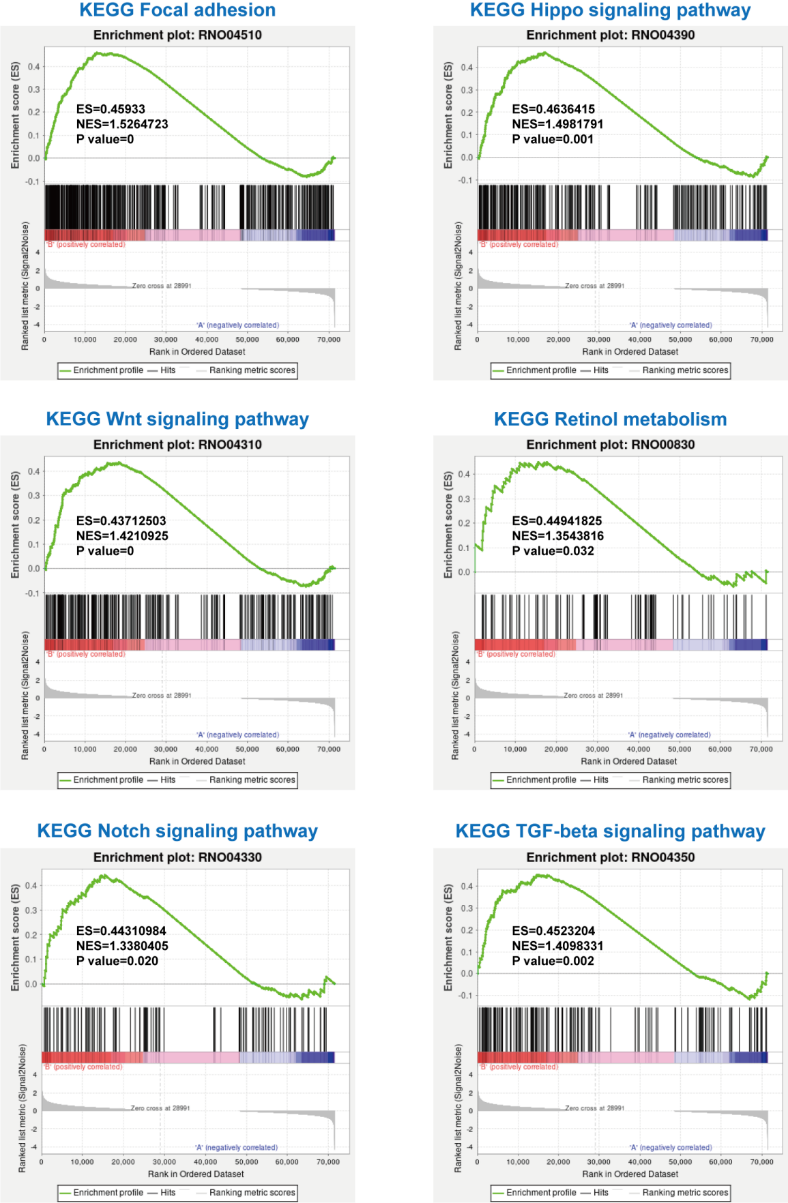
**

**Figure S11.** Significant GSEA plots (KEGG) related to chondrogenesis and osteogenesis.

# References

1. Cao X, Yan B, Huang Y, Zhang Y, Li L, Qiu J, Lyu X. Use of laponite as adsorbents for Ni(II) removal from aqueous solution. *Environ. Prog. Sustain. Energy* **2017**;37:942-950.

2. Muyonga JH, Cole CGB, Duodu KG. Fourier transform infrared (FTIR) spectroscopic study of acid soluble collagen and gelatin from skins and bones of young and adult Nile perch (Lates niloticus). *Food Chem.* **2004**;86:325-332.

3. Kong J, Yu S. Fourier transform infrared spectroscopic analysis of protein secondary structures. *Acta Biochim. Biophys. Sin.* **2007**;39:549-559.

4. Frazier SD, Srubar WV. Evaporation-based method for preparing gelatin foams with aligned tubular pore structures. *Mater. Sci. Eng. C* **2016**;62:467-473.

5. Rathore P, Schiffman JD. Beyond the Single-Nozzle: Coaxial Electrospinning Enables Innovative Nanofiber Chemistries, Geometries, and Applications. *ACS Appl. Mater. Interfaces* **2020**;13:48-66.
